# Quantum simulation of 2D topological physics in a 1D array of optical cavities

**DOI:** 10.1038/ncomms8704

**Published:** 2015-07-06

**Authors:** Xi-Wang Luo, Xingxiang Zhou, Chuan-Feng Li, Jin-Shi Xu, Guang-Can Guo, Zheng-Wei Zhou

**Affiliations:** 1 Key Laboratory of Quantum Information, University of Science and Technology of China, Hefei, Anhui 230026, China.; 2 Synergetic Innovation Center of Quantum Information and Quantum Physics, University of Science and Technology of China, Hefei, Anhui 230026, China.

## Abstract

Orbital angular momentum of light is a fundamental optical degree of freedom characterized by unlimited number of available angular momentum states. Although this unique property has proved invaluable in diverse recent studies ranging from optical communication to quantum information, it has not been considered useful or even relevant for simulating nontrivial physics problems such as topological phenomena. Contrary to this misconception, we demonstrate the incredible value of orbital angular momentum of light for quantum simulation by showing theoretically how it allows to study a variety of important 2D topological physics in a 1D array of optical cavities. This application for orbital angular momentum of light not only reduces required physical resources but also increases feasible scale of simulation, and thus makes it possible to investigate important topics such as edge-state transport and topological phase transition in a small simulator ready for immediate experimental exploration.

## Abstract (web summary)

A wide variety of interesting phenomena arise in 2D systems subject to external gauge fields, but these are sometimes challenging to verify experimentally. Here the authors propose a setup to simulate 2D physics with a 1D arrangement of cavities, by exploiting the orbital angular momentum of trapped photons.

## Graphical abstract

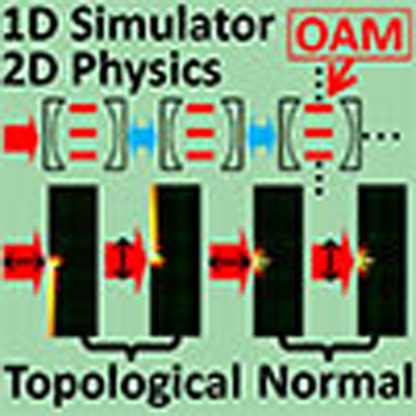

As a relatively under-exploited optical degree of freedom, orbital angular momentum (OAM) of light has motivated much exciting research lately. Beams of OAM-carrying photons have an azimuthal phase dependence in the form 
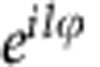
 where the OAM quantum number *l* can take any integer value^1^. These photon modes, which arise in the natural solutions of the paraxial wave equation in cylindrical coordinates^2^, can be manipulated and measured with high precision^3^,^4^,^5^,^6^. Because of the unlimited range of the angular momentum, OAM-carrying photons are recognized as a unique asset in many studies. On the application side, they are used to enable high-capacity optical communication^7^,^8^ and versatile optical tweezers^9^. In fundamental research, they have played important roles in quantum information and quantum foundation^6^,^10^,^11^,^12^,^13^,^14^,^15^. Although experimental study of OAM of light used to be limited to low angular momentum, there has been tremendous advance lately motivated by its great potential. This is highlighted by the remarkable recent demonstration of quantum entanglement involving angular momenta as high as hundreds^16^,^17^.

In spite of the many successful recent studies involving the OAM degree of freedom of light, its exploitation is still at an early stage and many novel possibilities remain unrecognized. In particular, it has not been considered useful for quantum simulation of important physics problems such as the extraordinary topological phenomena that arise in two-dimensional (2D) systems subject to external gauge fields. These include the likes of integer^18^ and fractional^19^ quantum Hall effect and quantum spin Hall effect^20^, which are characterized by exotic properties such as quantized conductance and edge-state transport. They are often difficult to investigate due to stringent experimental conditions required, and some theoretical predictions remain challenging to observe^20^,^21^. Because of this, various quantum simulation schemes based on different physical platforms such as ultracold atoms^22^,^23^,^24^ and photons^25^,^26^,^27^,^28^,^29^,^30^,^31^,^32^,^33^,^34^,^35^ have been suggested recently. None of them involves OAM of light whose connection to topological physics appears to be nothing but an illusion even in concept. Not surprisingly, central to most existing simulation schemes is a 2D architecture for the simulator. Many of them are still very demanding, requiring limit-pushing experimental conditions or advanced new technologies.

In this work, we show that it is not only possible, but advantageous to use the OAM of light for nontrivial quantum simulation by demonstrating theoretically how it can enable and support the study of a broad range of topological physics. In contrast to other proposals^25^,^26^,^27^,^28^,^29^,^30^,^31^,^32^,^33^, our system has a one-dimensional (1D) structure that does not need to be large in scale, thus reducing the complexity of the simulator. Feasible scale of simulation is increased despite the simplified system, and it is so versatile that the effect of arbitrary Abelian and non-Abelian gauge fields can be studied using standard linear optics devices only, with no restriction on the form of the gauge fields^29^,^30^,^33^ and no need for specially designed meta-material^31^ or photonic crystal^33^. It then allows to investigate important topological problems under intense pursuit such as non-Abelian gauge field induced phase transition between a photonic normal and topological insulator. Further, we can easily probe the topological properties of our system by measuring the photon transmission coefficients which are shown to have deep connections to the essential topological invariants of the system. All this is possible because of the inherent properties of the OAM of light.

## Results

### The 1D array of cavities

**Figure 1:**
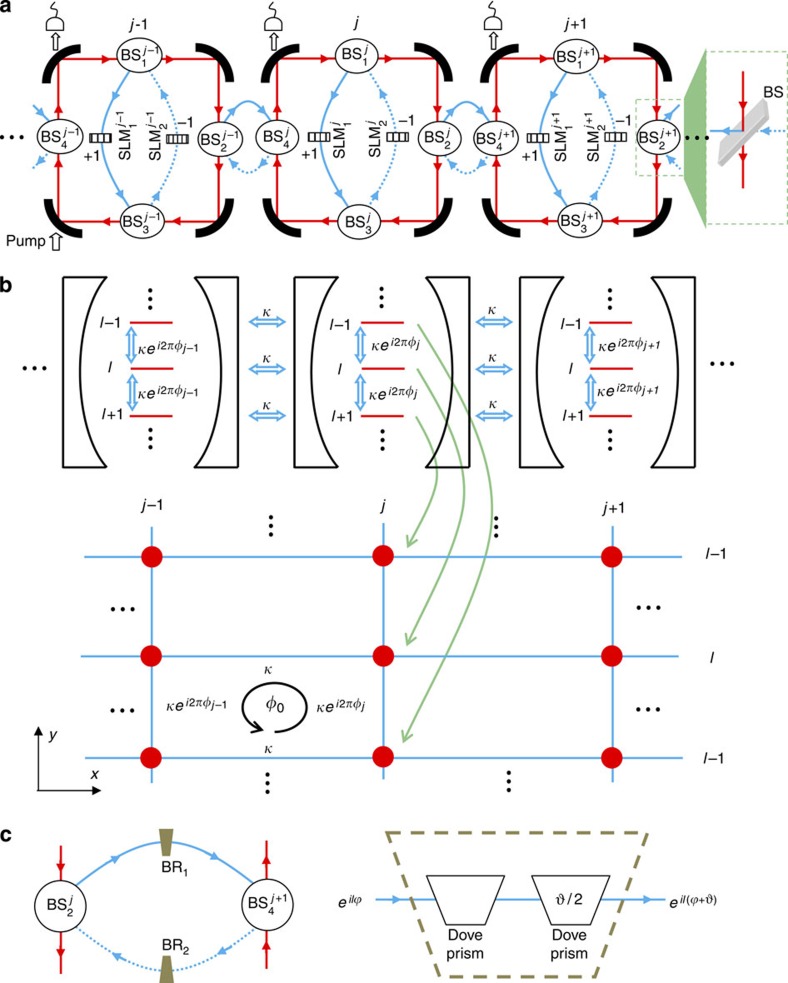
A 1D array of degenerate cavities for simulating a 2D rectangular lattice in a magnetic field. (**a**) The optical design for simulating 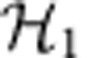. Each main cavity has an auxiliary cavity consisting of two BSs (
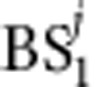
 and 
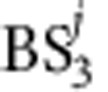
) and two SLMs (
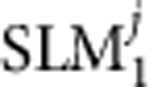
 and 
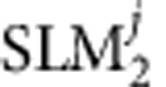
). There is also a coupling cavity (made of 
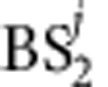
 and 
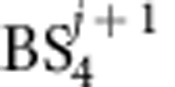
) between adjacent main cavities (It can be replaced with a simple BS to reduce the number of optical elements in experiments). The length of both the auxiliary and coupling cavity is chosen for destructive interference, and most light remains in the main cavity. The cavities at the two ends of the array can be coupled to realize periodic boundary condition, or uncoupled for open boundary condition. (**b**) Mapping of the 1D simulator array in (**a**) to a 2D rectangular lattice in a magnetic field. (**c**) The coupling cavity (left) for simulating 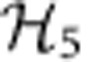 and the optical design (right) for the beam rotators BR_1_ and BR_2_ with opposite rotation angles ±*ϑ*=±2*πφ*_0_. The main cavity and auxiliary cavity require no modification, except that the phase difference between the arms containing the SLMs is set to 0.

Shown in Fig. 1a is our simulation system. It consists of an array of *N* nominally identical cavities that are coupled along the *x* direction. The system size, *N*, does not need to be large; we will show that even a simulator with just a few cavities is sufficient to demonstrate topological effects. The building blocks are degenerate cavities^36^,^37^, which have appropriate optical design such that they can support photon modes with different OAM (Supplementary Note 1). In each cavity, we make use of clockwise-circulating OAM-carrying photons and denote their annihilation operator 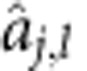, where *j* (0≤*j*≤*N*–1) is the index of the cavity in the array and *l* is the OAM number of the photon mode. To manipulate the OAM state of photons, for each cavity we introduce an auxiliary cavity consisting of two beam splitters (BSs) and two spatial light modulators (SLMs). The BSs divert a portion of the light in the main cavity towards the SLMs and merge it back. When propagating between the BSs, photons can accumulate a phase. The SLMs, which can be simple spiral phase plates with very low loss^38^,^39^, change the OAM of photons by ±1.

As depicted in Fig. 1b, by associating the OAM number of the photon in a cavity with the site index number along the *y* direction of an (imaginary) lattice, we can conceptually map our 1D array of cavities to a 2D rectangular lattice system. In Fig. 1a, the BSs and SLMs of the auxiliary cavity change the OAM of a portion of the light in the main cavity by ±1, and this corresponds to hopping of a particle on the lattice site in Fig. 1b along the *y* direction to its neighbouring sites with a probability determined by the reflectivity of the BSs. In this hopping process, the particle can also acquire an experimentally controllable phase determined by the imbalance between the optical paths from 
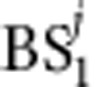
 to 
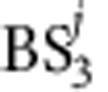
 and backwards. As shown in the Supplementary Note 2, the Hamiltonian of the simulated system is


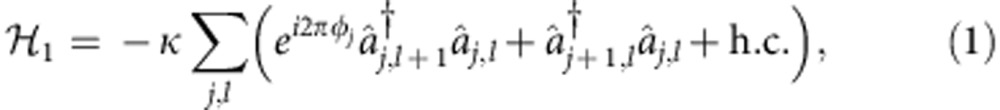


where *κ* is the transition rate between different OAM states, chosen to be the same with the coupling rate between neighbouring cavities, and 2*πφ*_*j*_ is the phase acquired by the photon in the *j*th cavity when it travels between the BSs in the auxiliary cavity. The term h.c. stands for Hermitian conjugate of the prior terms. If we set up the system such that *φ*_*j*_ is linearly dependent on the cavity index *j*, *φ*_*j*_=*jφ*_0_, then 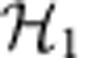 describes a tight-binding model of charged particle in a 2D lattice subject to a uniform magnetic field with *φ*_0_ quanta of flux per plaquette^40^.

**Figure 2:**
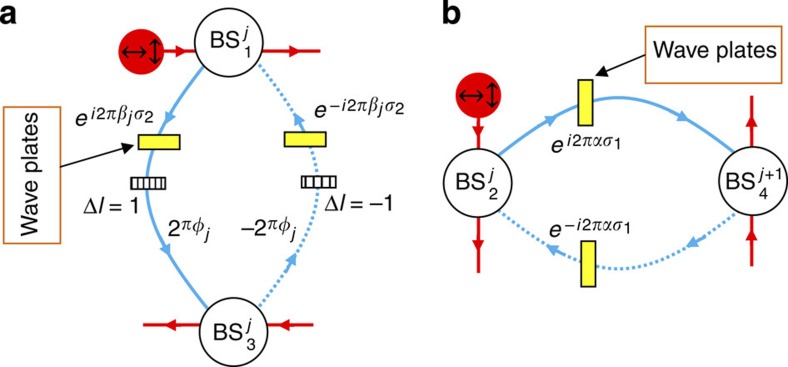
Design of the auxiliary and coupling cavity for simulating 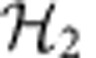 with polarized photons. (**a**) Two birefringent waveplates are inserted in the the auxiliary cavity. They can induce different phase delays for the two polarizations and cause transitions between them when their optical axes are properly aligned (Supplementary Note 3). Their effect is described by the Jones matrices 
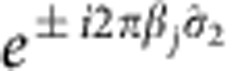
, where the two polarizations are represented by the spin up and down and the Pauli matrix 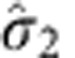 is determined by the orientation of the waveplate and its thickness. ±2*πφ*_*j*_ are the polarization-independent phase delays for the two optical paths. (**b**) Two waveplates with Jones matrices 
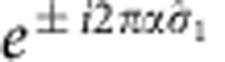
 are inserted in the coupling cavity to manipulate the polarization of the photon when it tunnels between adjacent main cavities.

Therefore, by representing a spatial degree of freedom with the OAM states of photons, we can study a 2D system with a 1D simulator, greatly reducing the physical resources required for the simulation. In contrast to earlier 1D optical simulator^34^, our system performs a full and genuine 2D simulation, rather than simulate the 1D behaviour of the system at a fixed Bloch momentum in the other direction. Meanwhile, in comparison with a 2D array of coupled cavities, the size of the 2D lattice that can be simulated is markedly increased along the *y* direction. This is due to the fact that, unlike in an atomic system^41^ where only a small number of atomic states are available for the simulation, there is no upper limit for the OAM of photons in theory. In reality, it is limited by practical factors such as the size of the optical elements and can be made very large in a proper design. In contrast, the feasible size in the *y* direction for a 2D cavity array would be much smaller, because nonuniformity of the cavities and local disturbances will make photons quickly lose coherence after travelling through a few cavities. This remarkable combination of reduced physical resources and increased scale of simulation makes our system very promising. Also, our system can be easily modified to support more demanding simulations by making use of additional degrees of freedom of photons. For instance, we can simulate the quantum spin Hall effect^42^ in non-Abelian gauge fields^43^,^44^ by using the horizontal and vertical polarizations of polarized photons to represent the up and down state (*s*=±1) of a spin. By using birefringent waveplates whose optical axes are properly aligned with respect to the horizontal and vertical polarizations, we can assign different phases to the two polarizations and cause transitions between them when they pass the waveplates (see Supplementary Note 3 for details). We can then manipulate the polarization states of the photon to mimick the spin flips and spin-dependent phase delays caused by non-Abelian gauge fields, as illustrated in Fig. 2. The simulated Hamiltonian is (Supplementary Note 3)


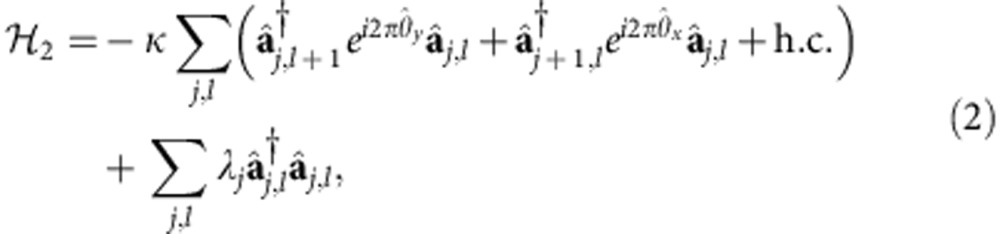


where 
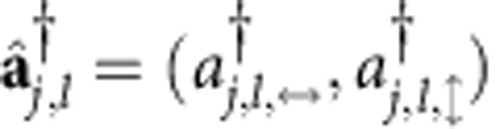
 is a two-component (the horizontal and vertical polarization) photon creation operator, and *λ*_*j*_ is an effective on-site energy. The tunnelling phases that correspond to the potentials of the associated gauge fields^22^, are given by


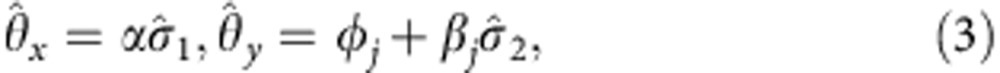


where *φ*_*j*_ is the spin-independent part of the phase, and *α*, *β*_*j*_, 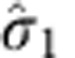 and 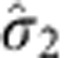 are determined by the Jones matrices^2^ of the waveplates as shown in Fig. 2. By selecting appropriate waveplates and manipulating the polarization of the photon accordingly, we can engineer non-commuting tunnelling phases 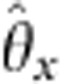 and 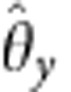, and thus simulate the effect of an arbitrary non-Abelian gauge field.

### Probing scheme

Since we represent a spatial degrees of freedom with OAM states of photons, the measurement of our system involves manipulation and detection of the OAM states. Specifically, we pump the *j*_i_th cavity using a probing light with a definitive OAM *l*_i_ and measure in the steady state how much ends up in the OAM mode *l*_o_ in the *j*_o_th cavity by leaking a small amount of light out of each cavity, as shown in Fig. 1a. It is determined by the transmission coefficient^45^ (Supplementary Note 4)


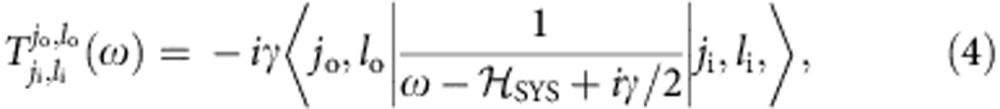


where *ω* is the detuning of the probing light from the cavity frequency, *γ* is the photon loss of the system, and 
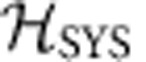
 is the simulated Hamiltonian. When non-Abelian gauge fields are concerned, the polarization indexes *s*_i_ and *s*_o_ should also be included for the input and output modes.

Generation and detection of OAM-carrying photons can be accomplished very reliably^3^,^6^. By a coherent measurement, we can determine both the amplitude and phase of 
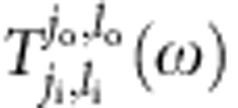
. Thanks to the 1D structure of our system and the use of OAM states, we can perform this measurement between any pair of (*j*_i_, *l*_i_) and (*j*_o_, *l*_o_), equivalent to measuring the transmission coefficient between any pair of sites in the simulated 2D lattice. Such powerful probing capability is key to the demonstration of various topological effects in our system.

Feasible measurement and clear demonstration of topological properties is the topic of many recent studies^21^,^31^,^32^,^46^,^47^,^48^ since generally speaking it is a very challenging task. Remarkably, in our system it is straightforward and requires no more than measuring the photon transmission coefficient in equation (4). As we will show, there is a deep connection between the photon transmission coefficient and the essential topological invariants, which can be exploited to demonstrate topological behaviour in optical systems.

### System spectrum and density of states

**Figure 3:**
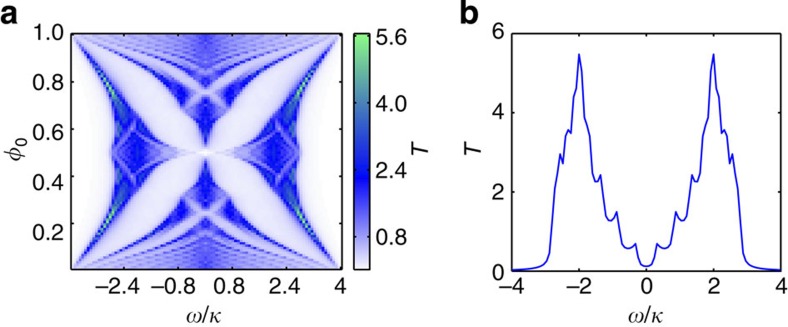
Simulation of photon transmission spectroscopy. (**a**) Calculated transmission spectra 
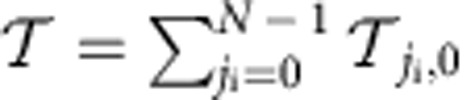
 of 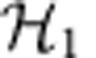 under different values of magnetic flux *φ*_0_. Since it is possible to measure the transmission coefficient between every pair of lattice sites, we add transmissions to all output channels to obtain a strong signal and increase the sensitivity of the measurement. (**b**) Calculated transmission spectrum 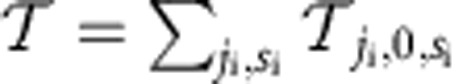 of 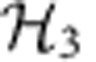, where 
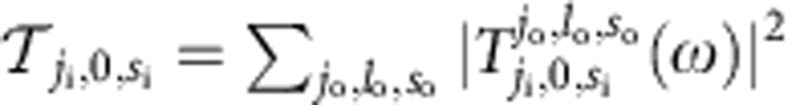
. In both **a** and **b**, the size of the simulator *N*=10. The OAM number of the photon included in the calculation is *l*∈[−50, 50]. Open and periodic boundary conditions are used in the *x* and *y* direction. The photon loss *γ*=0.1*κ*.

As can be seen in equation (4), 
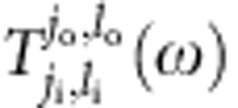
 is sensitive to the energy mismatch between the frequency of the probing light and the energy of the system. Because of this, we can study the system's spectrum by measuring the transmission coefficient


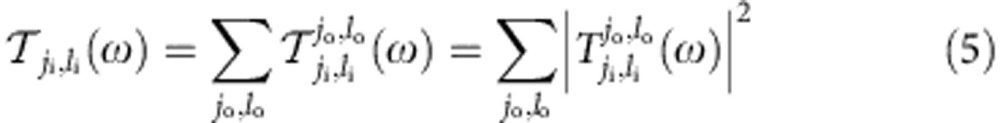


as a function of the frequency of the probing light, where 
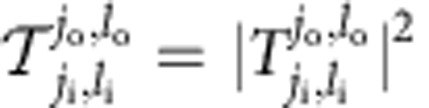
. For a system in an Abelian gauge field described by 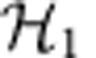, we calculate and plot in Fig. 3a the system spectrum which is the well-known Hofstadter butterfly^40^. We see that the main characteristics of the system spectrum are clearly identifiable even in a small simulator with just a few cavities.

The transmission spectroscopy is also very valuable for studying physics associated with a non-Abelian gauge field. As an example, in equation (3), if we choose 
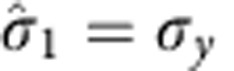
, 
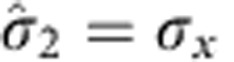
, 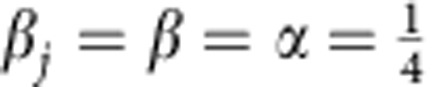, *λ*_*j*_=0, and *φ*_*j*_=*jφ*_0_=0, we get the 2D Dirac's Hamiltonian on a lattice^49^


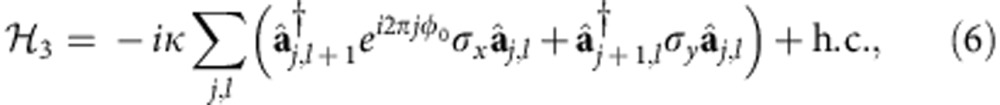


which is a topic of intense research because of its importance for understanding the properties of graphene and other exotic systems^23^,^24^,^50^,^51^. Characteristic of 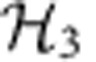 are four conical singularities at the Dirac points^51^ in the spectrum, which give rise to massless relativistic particles. As the energy deviates from the Dirac points, the change of the dispersion relation from relativistic to non-relativistic is revealed by the Van Hove singularities in the density of states. When the decay rate *γ* is small, the density of states can be inferred from the photon transmission spectrum which is shown in Fig. 3b. The Dirac point at *ω*=0 and two Van Hove singularities near *ω*=±2*κ* are observed, confirming Dirac physics related behaviour in the system.

### Edge states and topological protection

**Figure 4:**
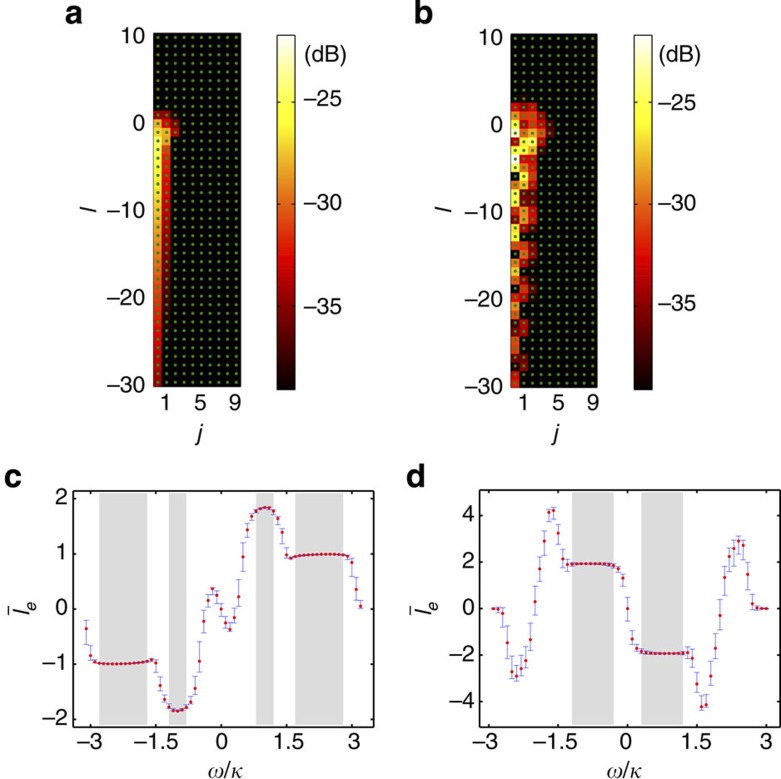
Simulation of edge-state transport. (**a**) Calculated photon transmission 
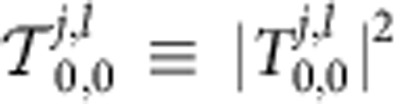
 for 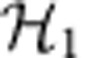 with *φ*_0_=1/6. The frequency of the probing light, *ω*=−2.2*κ*, is located in the middle of the first band gap. There is one-edge mode in this large band gap. (**b**) Calculated photon transmission when the probing light frequency *ω*=−1.0*κ* is located in the smaller second gap. Two-edge modes are present and interference patterns due to their phase velocity mismatch are observed. (**c**) Calculated average OAM displacement 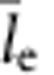 (red dots) for 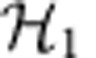 with *φ*_0_=1/6. Also shown is its s.d. indicated by the blue error bars. It is calculated by assuming a Gaussian distributed random shift *δλ* in the cavity resonance frequency with a s.d. *σ*(*δλ*)=0.1*κ*. The grey areas mark the frequency ranges of the band gaps. (**d**) 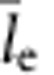 (red dots) and its s.d. (blue error bars) for 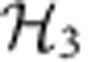 with *φ*_0_=1/20. In **a**–**d**, the size of the simulator *N*=10. The OAM included in the calculation is *l*∈[−50,50]. Open and periodic boundary conditions are used in the *x* and *y* direction. The photon loss is *γ*=0.1*κ* in **a** and **b**, and *γ*=0.2*κ* in **c** and **d**.

One of the most remarkable phenomena in topological physics is the existence of topologically protected chiral edge states in the band gaps of a finite lattice. In our system, we can study the edge states by pumping the cavity at the end of the 1D simulator array using a probing light beam with a definitive OAM. It is equivalent to driving a site on the edge of a 2D lattice. When the frequency of the probing light falls in a band gap, excitation of gapless edge states dictates that the light can only propagate along the edge of the simulated system. This is clearly demonstrated in Fig. 4a,b, where chiral edge-state transport is observed in a small simulator.

To study the robustness of the edge states against disorder, we introduce the average OAM ‘displacement' for the transport process defined as


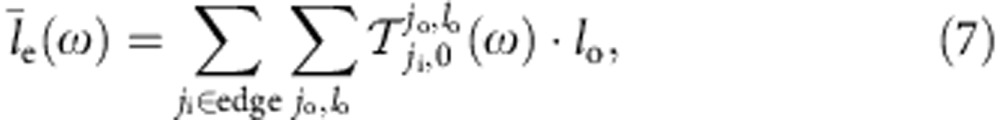


where 
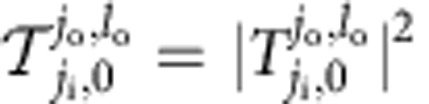
, and 
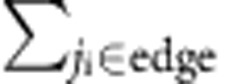
 refers to summation over the sites close to one edge of the lattice where the amplitude of the edge states is significant. As proved in the Supplementary Note 5, when the frequency of the probing light *ω* falls in a large band gap, 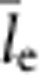 has the interesting property that it is equal to the total Chern number for the bands below the gap. Also, the value of 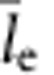 is mainly determined by states roughly in resonance with *ω*. Consequently, how 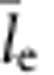 is disturbed by disorder is a measure for the robustness of these states. Shown in Fig. 4c are 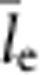 and its variation caused by a random shift in the cavity resonance frequency. It can be concluded that the edge states are almost immune to the disorder when the band gap is large compared with the photon loss and random cavity frequency shift, whereas the in-band states are strongly affected.

In addition to its fundamental interest, edge-state transport is also very useful for probing the topological behaviour of a system. One such example is the observation of the relativistic quantum Hall effect which arises in the Dirac Hamiltonian 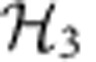 with small but nonzero magnetic flux *φ*_0_. As shown in Fig. 4d, 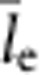 experiences a double-step leap from 2 to −2 around the Dirac point at *ω*=0 caused by a sudden change in the topological property of the system. Such exotic behaviour^43^,^44^ was predicted and observed in graphene^52^,^53^.

### Topological quantum phase transition

By measuring the system spectrum and edge-state transport, we can study nontrivial physics such as topological quantum phase transitions driven by non-Abelian gauge fields, which are important for understanding novel quantum states of matter such as topological insulators and superconductors^21^,^23^,^24^,^43^,^44^,^54^,^55^. In our system with non-Abelian gauge field, if we choose 
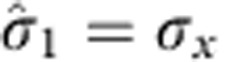
, 
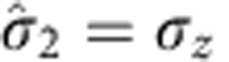
, *φ*_*j*_=0, *α*=1/4, *β*_*j*_=*j*/4+*β*_0_ and *λ*_*j*_=*λ*_0_·[mod(*j*, 4) – 1.5] in equation (3), the Hamiltonian in equation (2) becomes


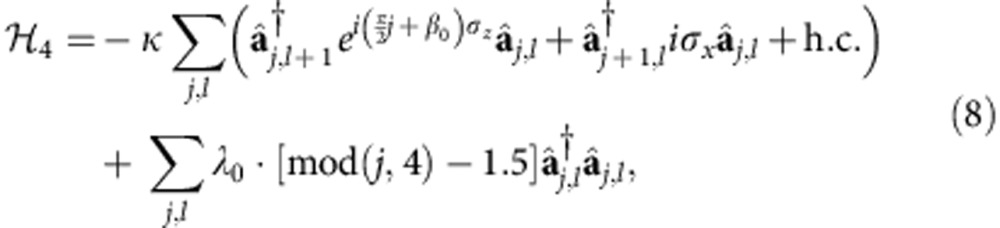


which describes an effective spin in a non-Abelian gauge field characterized by spin-dependent magnetic field and strong spin–orbit coupling. Also present is a periodically modulated on-site potential *λ*_*j*_. In the simulation system, the horizontal and vertical polarizations with degenerate on-site energies flip to their counterpart when the photon tunnels between cavities and acquire opposite phases when the photon goes around a plaquette in the simulated lattice in the same direction. This is the same behaviour with that of the spin up and down in an electronic system, which has time-reversal symmetry, and polarized photon-edge states analogous to spin edge states can emerge in our system. The two polarized edge states are associated with opposite Chern numbers, and thus their total Chern number *C* is 0 whereas the difference 2*ν* can be nonzero. The properties of such a photonic topological insulator are in contrast with those of a normal insulator in which both *C* and *ν* are 0 and photon transport of both polarizations is strongly suppressed.

**Figure 5:**
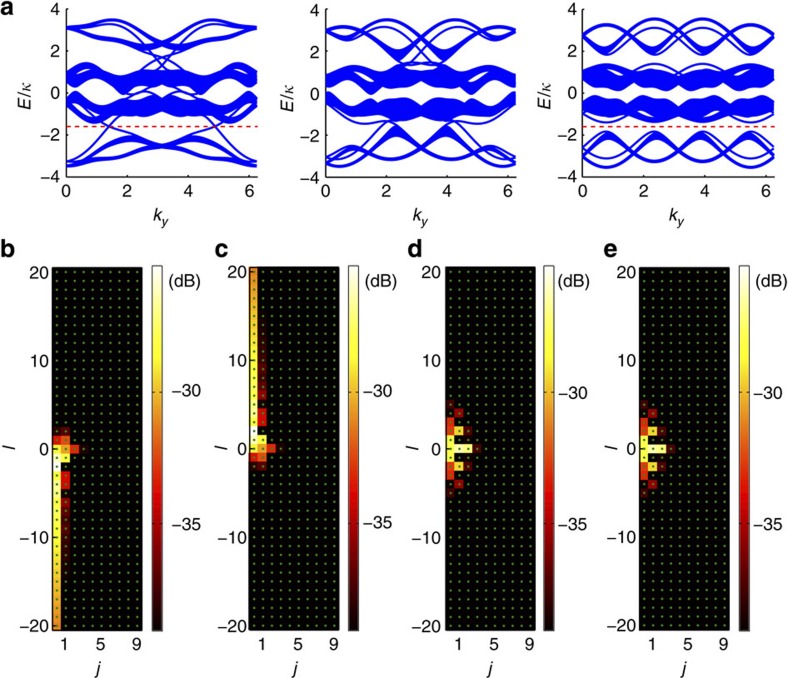
Simulated topological quantum phase transition and edge-state transport. (**a**) Calculated band structure for 
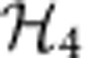
 with *λ*_0_=0.6*κ*. The value of *β*_0_ is 0, 0.075, and 0.125 from left to right. (**b**) Calculated photon transmission 
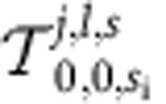
 when *β*_0_=0. The first cavity in the simulator array is pumped by a probing light with *l*_i_=0 and *s*_i_=↔. The frequency of the probing light *ω*=−1.6*κ* is located in the first band gap. (**c**) The same as in **b**, except that the polarization of the probing light is changed to the other value, *s*_i_=↕. (**d**) The same as in **b**, except that *β*_0_=0.125. (**e**) The same as in **c**, except that *β*_0_=0.125. In (**b**–**e**), the size of the simulator *N*=10. The OAM included in the calculation is *l*∈[−50,50]. Open and periodic boundary conditions are used in the *x* and *y* direction. The photon loss rate is *γ*=0.1*κ*.

A topological quantum phase transition can be induced in the system by adjusting the value of the non-Abelian gauge field. In Fig. 5a, it is shown how the band structure of the system changes with *β*_0_. As *β*_0_ increases, the first band gap near *ω*=−1.6*κ* closes and opens again. Initially, when *β*_0_ is small, the topological index *ν* of the system is *ν*=1, and the system is in a topological insulator state. Correspondingly, there are a pair of photon-edge states with opposite polarizations propagating in opposite directions as shown in Fig. 5b,c. These polarized-edge states are protected as long as the local noise does not disturb the symmetry between the two polarizations so that their on-site energies stay degenerate and their phases around a plaquette remain opposite to each other. When the energy gap opens again with a large *β*_0_, *ν* changes to 0, and the system becomes a normal insulator. This is confirmed by the disappearance of the photon-edge states in Fig. 5d,e.

### Measurement of the chern number

**Figure 6:**
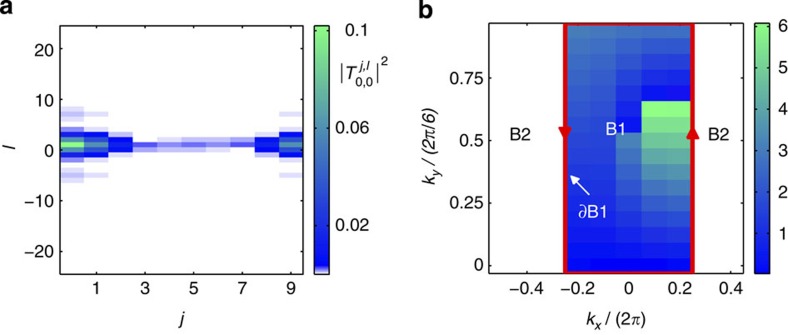
Simulation of the Chern number measurement for 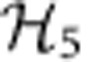. (**a**) Calculated photon transmission 
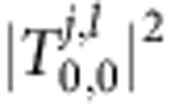
. The magnetic flux *φ*_0_=1/6. The probing light frequency is at *ω*=−3.09*κ*, where lies the very narrow first energy band. (**b**) Calculated phase mismatch *χ*(*k*_*x*_,*k*_*y*_) of 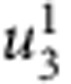 in the Brillouin zone resulting from two different phase conventions for 
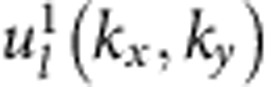
 in equation (10), defined by dividing the Brillouin zone into B1, whose boundary is marked by the red line and where 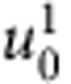 is always nonzero, and B2, which contains all zero points of 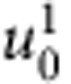 but where 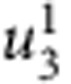 does not vanish. In one phase convention, 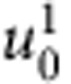 is real in B1. In the other convention, 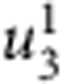 is real in B2. The Chern number is determined by the integration of the gradient of *χ*(*k*_*x*_, *k*_*y*_) on the boundary of B1, ∂B1 (Supplementary Note 6). In **a** and **b**, the size of the simulator *N*=10. The OAM included in the calculation is *l*∈[−48, 48). Periodic boundary conditions are used in both the *x* and *y* directions. The photon loss rate *γ*=0.1*κ*.

The Chern number is the ultimate quantum invariant to classify topological states and characterize their behaviour^21^. As shown in Fig. 4c, in a finite lattice the Chern number can be measured via the average OAM displacement 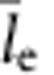 for edge-state transport. In an infinite system, the Chern number is equivalent to the TKNN index^56^. For its measurement, we insert a pair of beam rotators (BRs) with opposite rotation angles ±*ϑ*=±2*πφ*_0_ in the coupling cavities, as shown in Fig. 1c. A BR with a rotation angle *ϑ* is made of two Dove prisms rotated by *ϑ*/2 with respect to each other and can change the azimuthal dependence of the OAM mode from *e*^*ilφ*^ to *e*^*il*(*φ*+*ϑ*)^. We also balance the two paths of the auxiliary cavities containing the SLMs. The simulated Hamiltonian becomes


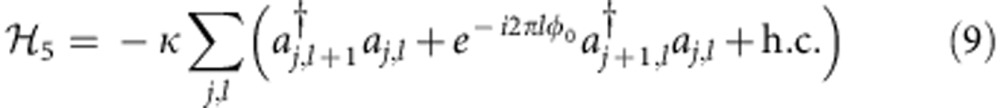


which is related to 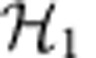 by a gauge transformation and helps keep the size of the simulator small (Supplementary Note 2). In Fig. 6a, the amplitude of the photon transmission coefficients 
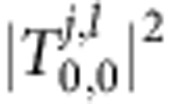
 is shown for a system with a rational magnetic flux *φ*_0_=1/6. Since the first energy band of this system is very narrow (see Supplementary Note 6), in a lossy cavity the probing light will be in resonance with the entire first energy band^57^. This allows us to determine the in-band Bloch eigenstates


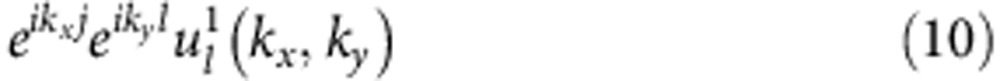


from the Fourier transforms of 
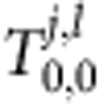
, where *k*_*x*_∈[−*π*,*π*], *k*_*y*_∈[0,2*π*/6] define the Brillouin zone and 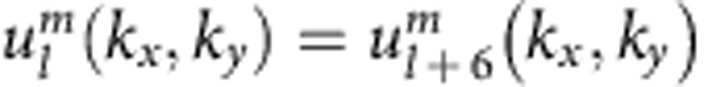 for the *m*th band is a periodic function. There is a Chern-number-conserving gauge freedom in the phase choices of 
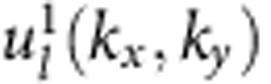
, as shown in Fig. 6b. *χ*(*k*_*x*_,*k*_*y*_), the phase mismatch of 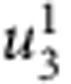 resulting from the two different phase conventions in Fig. 6b, can be used to calculate the Chern number (Supplementary Note 6). Our numerical calculation using *χ*(*k*_*x*_,*k*_*y*_) yields the Chern number 1 for the related band.

## Discussion

By mapping the OAM states of photons to spatial coordinates in a lattice, we have found a promising scheme for studying nontrivial 2D topological physics in a 1D physical simulator. Our method relies on only linear optics and manipulation of OAM states, and thus it can be realized with any physical systems that provide these elements or their equivalent, though longer wavelengths may have an advantage in coupling a large number of cavities. Our system is ready for immediate experimental exploration, because the key elements in our scheme, such as reliable manipulation of photon modes with high angular momenta^4^,^16^, precise measurement of the OAM states^5^,^6^, design and operation of degenerate cavities^36^,^37^ and locking of multiple optical cavities^58^, have all been realized. Our idea may also be used to simulate 1D problems with OAM modes in a single cavity^59^,^60^,^61^, and it can lead to novel photonic effects with practical applications^25^. Above all, by demonstrating the counter-intuitive application of photonic OAM in quantum simulation, our work deepens our understanding of the OAM degree of freedom and advances our view of photonic quantum simulation. Building on the presented ideas, we can then leverage the extreme flexibility and reliability in the design and operation of optical circuits for quantum simulation of various topological problems. All these issues and possibilities provide exciting opportunities for further investigation.

## Additional information

**How to cite this article:** Luo, X.-W. *et al*. Quantum simulation of 2D topological physics in a 1D array of optical cavities. *Nat. Commun.* 6:7704 doi: 10.1038/ncomms8704 (2015).

## Supplementary Material

Supplementary Information Supplementary Figures 1-6, Supplementary Notes 1-6 and Supplementary References
