# Supplementary material for: Quantum simulation of 2D topological physics in a 1D array of optical cavities
Source: Nat Commun. 2015 Jul 6;6:7704. doi: 10.1038/ncomms8704 (PMC4506549; doi:10.1038/ncomms8704)
Supplement: Supplementary Information — Supplementary Figures 1-6, Supplementary Notes 1-6 and Supplementary References [file ncomms8704-s1.pdf]

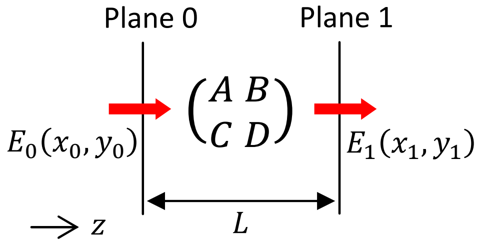

Supplementary Figure 1: Propagation of the light field between two planes perpendicular to the optical axis in a cavity.

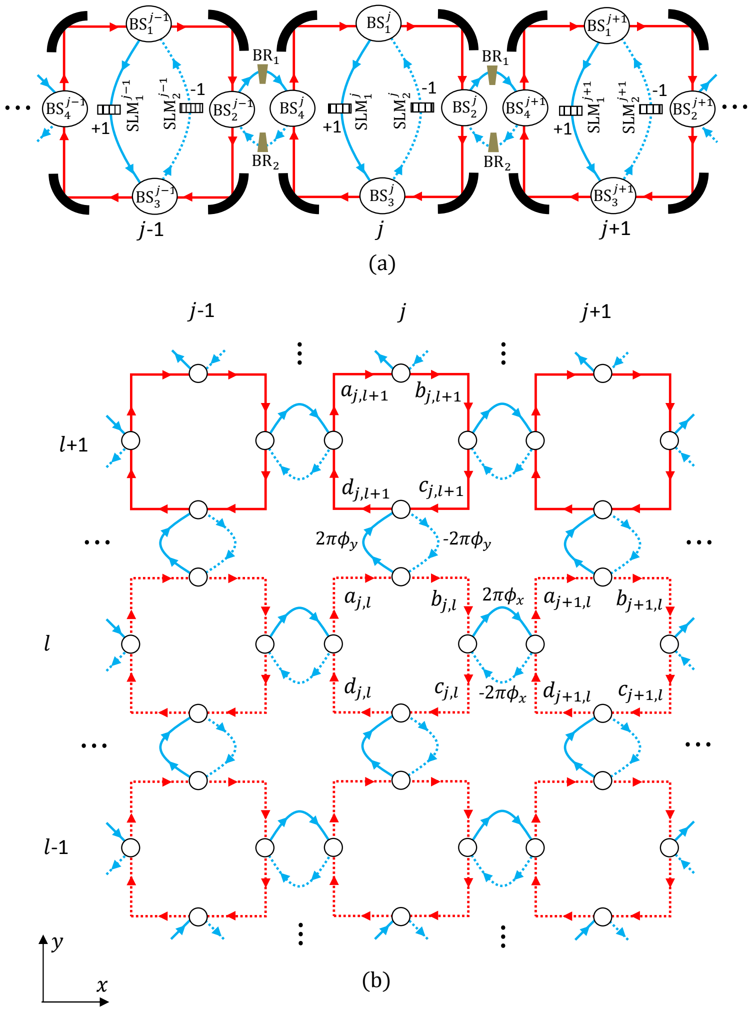

Supplementary Figure 2: (a) The simulator consisting of a *1d* array of cavities. Adjacent cavities are coupled by beam splitters (BSs). The SLMs in each cavity change the OAM number of the photon by ±1. The beam rotators BR_1 and BR_2, which have opposite rotation angles, can be use to implement a gauge transformation of the magnetic field (see Supplementary Note 2). Their detailed design is shown in Supplementary Figure 3. (b) The simulated *2d* lattice system. For the convenience of discussion, it is assumed that the BSs are placed at equal distances along the optical path of the main cavity. The field amplitudes $a_{j,l}$, $b_{j,l}$, $c_{j,l}$ and $d_{j,l}$ are defined at the mid point between adjacent pairs of BSs. $2\pi\phi_x$ $(2\pi\phi_y)$ is the phase imbalance between the two arms of the corresponding cavity.

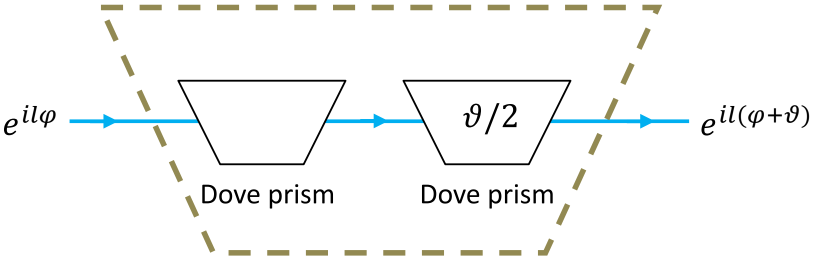

Supplementary Figure 3: A beam rotator consisting of two Dove prisms which are rotated by $\vartheta/2$ with respect to each other. Since a Dove prism flips the transverse profile of any transmitted beam, the two Dove prisms in the figure will rotate a propagating beam by an angle ϑ. It then changes the azimuthal phase dependence of the l-th OAM mode from $e^{il\varphi}$ to $e^{il(\varphi+\vartheta)} = e^{il\vartheta}e^{il\varphi}$.

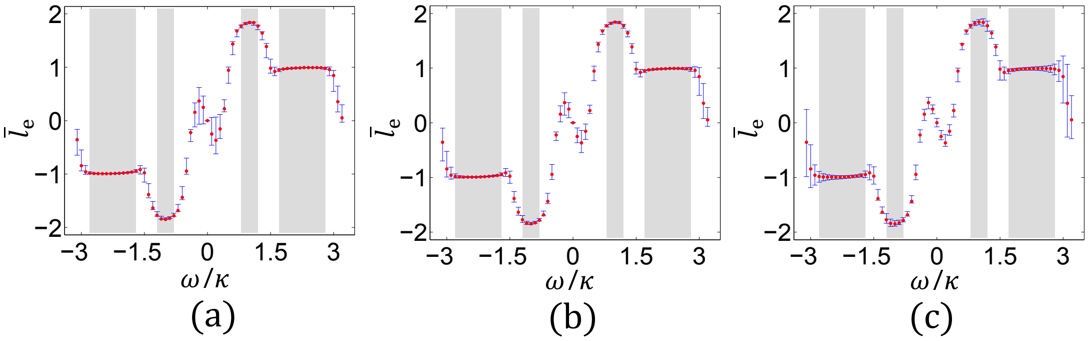

Supplementary Figure 4: The calculated average OAM displacement (red dots) for the photon transmission and its standard deviation (blue error bars) as a result of errors in coupling strengths and photon loss for $\mathcal{H}_1$. The magnetic flux $\phi_0 = 1/6$. The grey areas mark the frequency range of the band gaps. (a) Uncertainties in both the magnitude and phase of κ in the x direction are considered. They are assumed to have a Gaussian distribution with a standard deviation of $\Delta|\kappa| = 0.05|\kappa|$ and $\Delta\phi_\kappa = 0.05$rad. (b) OAM dependent uncertainties are considered, by assuming an error of $\delta|\kappa| \cdot F(l+\frac{1}{2})$ in the coupling between the l and $l+1$ mode and an error of $\delta\gamma \cdot F(l)$ in the photon loss for the l mode, where $F(x) = 1 - e^{-(\frac{x}{30})^2}$ and the uncertainties have Gaussian distributions with standard deviations of $\Delta|\kappa| = 0.05|\kappa|$ and $\Delta\gamma = 0.02\gamma$. (c) Independent uncertainties in couplings between OAM modes and photon loss for each cavity are considered. The result is averaged over input light with OAM number up to ± 3. The distribution and standard deviations of the uncertainties are the same as in (b). In (a)-(c), the size of the simulator $N = 10$. The OAM included in the calculation is $l \in [-50, 50]$. Open and periodic boundary conditions are used in the x and y direction. The photon loss rate is $\gamma = 0.2\kappa$.

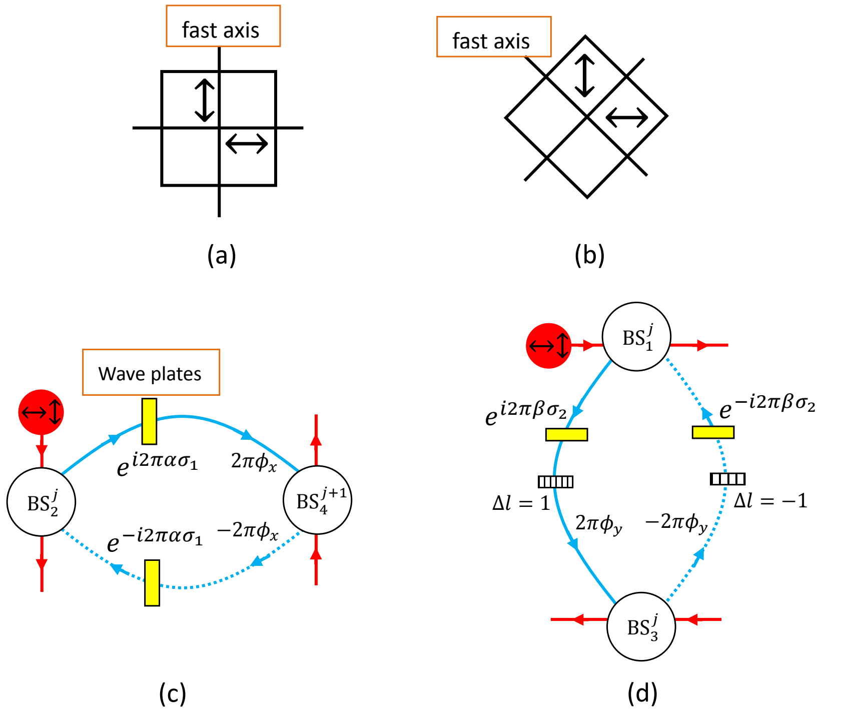

Supplementary Figure 5: (a) A waveplate whose fast axis aligns with the vertical polarization of the incident light. The Jones matrix is $e^{i2\pi\phi\sigma_z}$ with the phase $2\pi\phi$ is dependent on the thickness of the waveplate. (b) A waveplate whose fast axis is rotated by 45° with respect to the vertical polarization of the incident light. The Jones matrix is $e^{i2\pi\phi\sigma_x}$. (c) The coupling cavity in the x direction. The waveplates are designed to realize Jones matrices $e^{\pm i2\pi\alpha\sigma_1}$, where $\sigma_1 = \vec{\sigma}\cdot\mathbf{n_1}$ with $\mathbf{n_1}$ an arbitrary unit vector. $\pm 2\pi\phi_x$ is the spin-independent phase imbalance. (d) The auxiliary cavity with the SLMs to change the OAM number of the photons. The waveplates are designed to realize Jones matrices $e^{\pm i2\pi\beta\sigma_2}$, where $\sigma_2 = \vec{\sigma}\cdot\mathbf{n_2}$ with $\mathbf{n_2}$ an arbitrary unit vector. $\pm 2\pi\phi_y$ is the spin-independent phase imbalance.

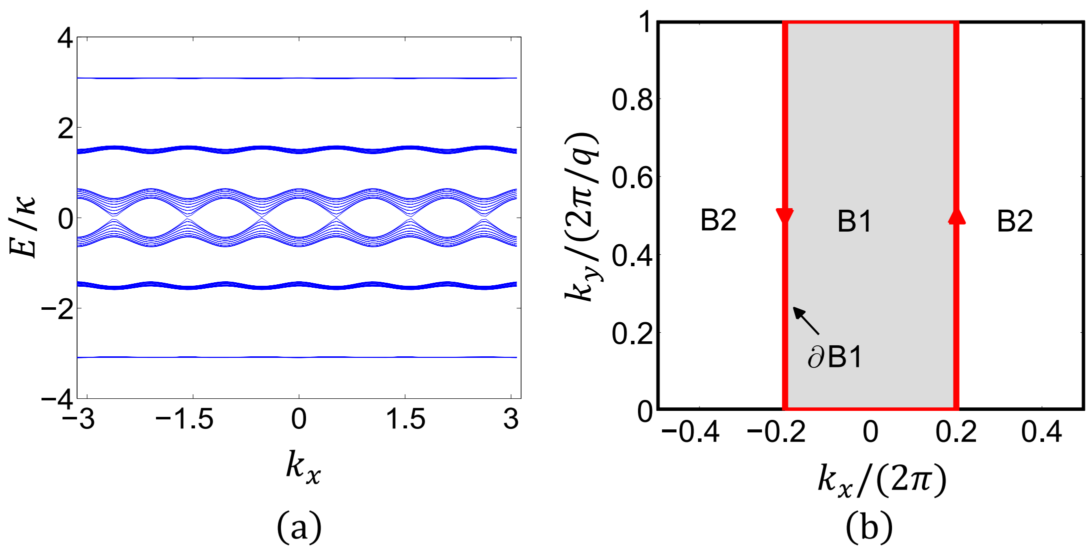

Supplementary Figure 6: (a) The energy band structure of $\mathcal{H}_2$ in Eq. (23) with a magnetic flux $\phi_0 = 1/6$. At each k_x, the eigenenergies for all possible values of k_y are calculated and plotted. It is seen that the bands around $E = \mp 3.09\kappa$ are very narrow (they contain all eigenenergies and are not a single line as they appear to be in the figure), with a width much less than 0.1κ. (b) Division of the magnetic Brillouin zone for the first band ($m = 1$) around $E = -3.09\kappa$. B1 is the area $\{k_x \in [-0.4\pi, 0.4\pi], k_y \in [0, 2\pi/6]\}$. Its boundary is marked by the red line. The rest is B2.

Supplementary Note 1: OAM modes in degenerate optical cavities

All optical cavities in our simulation system are degenerate cavities that can support optical modes with different orbital angular momentum (OAM). To understand the design principles of such cavities, we consider propagation of the light field in a cavity between two planes perpendicular to the optical axis as depicted in Supplementary Figure 1. For a cavity made of optical elements with rotational symmetry, under the paraxial approximation, the position and slope of a ray at the two planes, $[r_0, \dot{r}_0]^T$ and $[r_1, \dot{r}_1]^T$, are related by [1]

$$\begin{bmatrix} r_1 \\ \dot{r}_1 \end{bmatrix} = M \begin{bmatrix} r_0 \\ \dot{r}_0 \end{bmatrix} = \begin{bmatrix} A & B \\ C & D \end{bmatrix} \begin{bmatrix} r_0 \\ \dot{r}_0 \end{bmatrix}, \tag{1}$$

where the ray transfer matrix M between the two planes is determined by the optical design of the cavity. The electric fields at the two planes are also related by the Collins integral [2]

$$e^{-ikz_1} E_1(x_1, y_1) = e^{-ikL} e^{-ikz_0} \frac{i}{\lambda B} \int\int E_0(x_0, y_0) \exp[-\frac{i}{\lambda B}(Ax_0^2 + Dx_1^2 - 2x_0x_1 + Ay_0^2 + Dy_1^2 - 2y_0y_1)] dx_0 dy_0, \tag{2}$$

where λ and k are the wavelength and wave number, and L is the length of the optical path along the optical axis between the two planes.

The resonance frequencies and eigenmodes of the cavity can be solved for by using the condition that the field must reproduce itself after a round trip in the cavity. If the optical elements have cylindrical symmetry, the solutions are the Laguerre-Gaussian (LG) modes $E_{p,l}(r, \varphi)e^{-ikz}$ [1] with the transverse field

$$\begin{aligned} E_{p,l}(r, \varphi) = E_0 \frac{W_0}{W(z)} \left(\frac{r\sqrt{2}}{W(z)} \right)^{|l|} \mathcal{L}_p^{|l|} \left(\frac{2r^2}{W(z)^2} \right) \\ \times \exp \left(\frac{-r^2}{W(z)^2} \right) \exp \left(\frac{-ikr^2}{2R(z)} \right) \\ \times \exp \left[i(2p + |l| + 1)\zeta(z) \right] e^{il\varphi}, \end{aligned} \tag{3}$$

where $W(z) = W_0\sqrt{1 + (z/z_0)^2}$ is the transverse width of the light beam, $R(z) = z[1 + (z_0/z)^2]$ is the wavefront curvature radius, $\zeta(z) = \arctan(z/z_0)$ is the Gouy phase with beam waist W_0 and Raleigh range $z_0 = \pi W_0^2/\lambda$, and $\mathcal{L}_p^{|l|}(x)$ is the generalized Laguerre polynomial. The radial and azimuthal mode index p and l determine the transverse distribution of the electric field, since $p + 1$ is the number of radial nodes and $2\pi l$ is the phase variation for a closed path around the beam center. The resonance frequency for each $E_{p,l}$ mode in a ring-type cavity is determined by [3]

$$kL_0 - (2p + l + 1) \arccos \frac{A + D}{2} = 2n\pi, \tag{4}$$

where n is an integer, L_0 is the length of the round-trip optical path, and A and D are diagonal elements in the round-trip ray matrix. The off-diagonal elements of the round-trip ray matrix, B and C, only affect the beam waist W_0 of the resonance modes.

It is seen from Eq. (4) that, generally speaking, different $E_{p,l}$ modes are non-degenerate even for the same mode number n. However, If the cavity is properly designed such that $A = D = 1$ and $B = C = 0$, the resonance frequency becomes independent of the radial and azimuthal mode index p and l. Such a cavity is called a degenerate cavity. It can support photon modes of different p and l simultaneously. The design requirement of degenerate cavities is well understood; both general rules and concrete examples can be found in the literature [3–5].

Since each photon in a light beam with an azimuthal phase dependence $e^{il\varphi}$ carries an OAM of $l\hbar$ [6], we can have photons with different OAM in a degenerate cavity. In our simulator shown in Supplementary Figure 2 (a), there are three types of cavities with different roles to form a *1d* periodic array. Their optical design is as follows.

1. The main cavity in the array. Its length is chosen for constructive interference, $kL_0 = 2n\pi$. The elements for the half round-trip ray matrix of the optical paths $\mathrm{BS}_4^j \to \mathrm{BS}_1^j \to \mathrm{BS}_2^j$ and $\mathrm{BS}_2^j \to \mathrm{BS}_3^j \to \mathrm{BS}_4^j$ in Supplementary Figure 2 are $A = D = -1$, $B = C = 0$.

2. The coupling cavity between two adjacent main cavities consisting of BS_2^j and BS_4^{j+1}. Its length is chosen for destructive interference, $kL_0 = (2n + 1)\pi$. The elements of the ray matrix for the optical paths $\mathrm{BS}_2^j \to \mathrm{BS}_4^{j+1}$ and $\mathrm{BS}_4^{j+1} \to \mathrm{BS}_2^j$ are $A = D = -1$, $B = C = 0$.

3. The auxiliary cavity consisting of the two beam splitters BS_1^j, BS_3^j and the two spatial light modulators SLM_1^j, SLM_2^j. Its length is chosen for destructive interference, $kL_0 = (2n+1)\pi$. The elements of the ray matrix for optical paths $\mathrm{SLM}_1^j \to \mathrm{BS}_3^j \to \mathrm{SLM}_2^j$ and $\mathrm{SLM}_2^j \to \mathrm{BS}_1^j \to \mathrm{SLM}_1^j$ are $A = D = -1$, $B = C = 0$.

Supplementary Note 2: The tight-binding Hamiltonian

Derivation of the Hamiltonian

As explained in the main text, the *1d* simulator in Supplementary Figure 2 (a) is conceptually equivalent to the *2d* rectangular lattice in Supplementary Figure 2 (b). In order to derive the Hamiltonian of the simulated system, we consider the eigenmode field E which satisfies the Maxwell equation

$$\nabla \times (\nabla \times E) = \epsilon(\mathbf{r}) \frac{\omega^2}{c^2} E, \tag{5}$$

where $\epsilon(\mathbf{r})$ is the dielectric constant of the system and ω is the eigenenergy.

Under the assumption of weak coupling between cavities, E can be expanded in local modes (Wannier modes) [7–9],

$$E = \sum_{j,l} \psi_{j,l} W_{j,l}(\mathbf{r}), \tag{6}$$

where j is the index of the cavity in the simulator array and l is the OAM number of the photon. $W_{j,l}$, the Wannier mode localized at site (j,l), satisfies the Maxwell equation

$$\nabla \times (\nabla \times W_{j,l}) = \epsilon_0(\mathbf{r} - \mathbf{R}_{j,l}) \frac{\omega_0^2}{c^2} W_{j,l} \tag{7}$$

and is normalized to unity according to

$$\int d\mathbf{r} \epsilon_0(\mathbf{r} - \mathbf{R}_{j,l}) W_{j,l}^* W_{j,l} = 1 \tag{8}$$

with $\epsilon_0(\mathbf{r} - \mathbf{R}_{j,l})$ the dielectric constant at site (j,l), ω_0 the single-site resonance frequency, and $\mathbf{R}_{j,l} = j\hat{\mathbf{x}} + l\hat{\mathbf{y}}$ the lattice vector at site (j,l).

Using Eqs. (5), (6), and (7), we obtain

$$-\sum_{j',l'} \kappa_{j,l;j',l'} \psi_{j',l'} = (\omega - \omega_0)\psi_{j,l}, \tag{9}$$

where

$$\kappa_{j,l;j',l'} = \int d\mathbf{r} \frac{\omega_0}{2} [\epsilon(\mathbf{r}) - \epsilon_0(\mathbf{r} - \mathbf{R}_{j',l'})] W_{j,l}^* W_{j',l'}. \tag{10}$$

In deriving Eq. (9), we have used the weak coupling condition $(\omega - \omega_0)/\omega_0, \kappa_{j,l;j',l'}/\omega_0 \ll 1$, and kept only leading-order terms in $(\omega - \omega_0)/\omega_0$ and $\kappa_{j,l;j',l'}/\omega_0$. The on-site energy shift term $\kappa_{j,l;j,l}$ and non-adjacent coupling terms are usually negligibly small compared to the coupling term between adjacent cavities ($\kappa_{j,l;j+1,l}$ and $\kappa_{j,l;j,l+1}$), and we will drop them.

In Eq. (10), the integration is limited to the region where Wannier functions of neighboring cavities have appreciable overlap. In our system, it is on the beam splitters that couple the cavities. Also, the phase of the tunneling coefficient $\kappa_{j,l;j',l'}$ is sensitive to the phase of the Wannier functions. We can see that, when there is a phase imbalance $2\pi\phi_x$ between the two arms ($\mathrm{BS}_2^j \to \mathrm{BS}_4^{j+1}$ and $\mathrm{BS}_4^{j+1} \to \mathrm{BS}_2^j$) in the coupling cavity in Supplementary Figure 2 (a), the phase shift of the Wannier function in the integration region with respect to the balanced case $\phi_x = 0$ results in the relation

$$\kappa_{j,l;j+1,l}(\phi_x) = \kappa_{j,l;j+1,l}(0) e^{i2\pi\phi_x}, \tag{11}$$

where $\kappa_{j,l;j+1,l}(0)$ is the tunneling coefficient for the balanced case. Likewise, when the phase imbalance between the

two paths ($\mathrm{BS}_1^j \to \mathrm{SLM}_1^j \to \mathrm{BS}_3^j$ and $\mathrm{BS}_3^j \to \mathrm{SLM}_2^j \to \mathrm{BS}_1^j$) in the auxiliary cavities in Supplementary Figure 2 (a) is $2\pi\phi_y$, we have

$$\kappa_{j,l;j,l+1}(\phi_y) = \kappa_{j,l;j,l+1}(0)e^{i2\pi\phi_y}, \tag{12}$$

where $\kappa_{j,l;j,l+1}(0)$ is the tunneling coefficient in the y direction for the balanced case $\phi_y = 0$. If we choose the same coupling strength in the x and y direction, and denote $\kappa_{j,l;j+1,l}(0) = \kappa_{j,l;j,l+1}(0) = \kappa$, Eq. (9) then leads to the following tight-binding Hamiltonian in the rotating frame defined by $\mathcal{H}_0 = \sum \omega_0 \hat{a}^\dagger_{j,l}\hat{a}_{j,l}$,

$$\mathcal{H} = -\ \kappa \sum_{j,l} \left(e^{i2\pi\phi_x}\hat{a}^\dagger_{j+1,l}\hat{a}_{j,l} + e^{i2\pi\phi_y}\hat{a}^\dagger_{j,l+1}\hat{a}_{j,l} + h.c.\right), \tag{13}$$

where $\hat{a}_{j,l}$ and $\hat{a}^\dagger_{j,l}$ are photon annihilation and creation operators at site (j,l). As discussed in the main text, if we choose $\phi_x = 0$, and ϕ_y to be linearly dependent on the index j of the cavity in the simulator array, $\phi_y = j\phi_0$, the corresponding Hamiltonian

$$\mathcal{H}_1 = -\ \kappa \sum_{j,l} \left(\hat{a}^\dagger_{j+1,l}\hat{a}_{j,l} + e^{i2\pi j\phi_0}\hat{a}^\dagger_{j,l+1}\hat{a}_{j,l} + h.c.\right) \tag{14}$$

describes a *2d* system in a magnetic field with ϕ_0 quanta of flux per plaquette.

In some simulations we wish to introduce an on-site potential term to the Hamiltonian. For this purpose, we can slightly detune the resonance frequency of the main cavity from ω_0. This results in the following additional term in the Hamiltonian,

$$\sum_{j,l} \lambda_j \hat{a}^\dagger_{j,l}\hat{a}_{j,l},$$

where $\lambda_j = \omega_j - \omega_0$ and ω_j the resonance frequency of the j-th main cavity.

Dependence of the tunneling coefficient on the BS reflectivity

In order to select optical elements with appropriate parameters in experiments, we need to understand how the tunneling coefficient κ in Eq. (13) depends on the reflectivity of the BSs. This can be accomplished by using the transfer matrix analysis [10]. In Supplementary Figure 2 (b), we introduce the photon field amplitudes $a_{j,l}$, $b_{j,l}$, $c_{j,l}$ and $d_{j,l}$ at each lattice site (j,l). We assume that the phase imbalances $2\pi\phi_x$ and $2\pi\phi_y$ are the same for all lattice sites. In this case, the system is periodic in both the x and y directions with a period of 1. According to the transfer matrix formalism and Bloch theorem [11, 12],

$$\begin{pmatrix} a_{j',l'} \\ b_{j',l'} \\ c_{j',l'} \\ d_{j',l'} \end{pmatrix} = \begin{pmatrix} a_{j,l} \\ b_{j,l} \\ c_{j,l} \\ d_{j,l} \end{pmatrix} \cdot e^{-i(j'-j)K_x\Lambda - i(l'-l)K_y\Lambda}, \tag{15}$$

where Λ is the unit spacing and K_x, K_y are the Bloch quasi-momenta.

Assuming the reflection and transmission coefficients of all the BSs are $r = i|r|$ and $t = |t|$ ($|r|^2 + |t|^2 = 1$), we can write their transfer matrix as

$$M_{\mathrm{BS}} = \begin{pmatrix} \frac{1}{-i|r|} & \frac{t}{i|r|} \\ \frac{t}{-i|r|} & \frac{1}{i|r|} \end{pmatrix}. \tag{16}$$

Since the photons acquire a phase when they propagate between the BSs, we have

$$\begin{pmatrix} a_{j+1,l} \\ d_{j+1,l} \end{pmatrix} = M_x \cdot \begin{pmatrix} b_{j,l} \\ c_{j,l} \end{pmatrix} \tag{17}$$

with the field transfer matrix in the x direction

$$M_x = \begin{pmatrix} e^{-ikS_c/8} & 0 \\ 0 & e^{ikS_c/8} \end{pmatrix} \cdot M_{\rm BS} \cdot \begin{pmatrix} e^{-i(kS_a/2+2\pi\phi_x)} & 0 \\ 0 & e^{i(kS_a/2-2\pi\phi_x)} \end{pmatrix} \cdot M_{\rm BS} \cdot \begin{pmatrix} e^{-ikS_c/8} & 0 \\ 0 & e^{ikS_c/8} \end{pmatrix}, \tag{18}$$

and similar expressions for M_y in the y direction. Here, k is the wave number, and S_c and S_a are the total optical path length of the main cavity and the coupling cavity. Using the Bloch relation in Eq. (15), we can derive the following equations for the field amplitudes at site (j, l),

$$\begin{pmatrix} a_{j,l} \\ d_{j,l} \end{pmatrix} = M_x \cdot \begin{pmatrix} b_{j,l} \\ c_{j,l} \end{pmatrix} \cdot e^{iK_x\Lambda}, \tag{19}$$

and

$$\begin{pmatrix} d_{j,l} \\ c_{j,l} \end{pmatrix} = M_y \cdot \begin{pmatrix} a_{j,l} \\ b_{j,l} \end{pmatrix} \cdot e^{iK_y\Lambda}. \tag{20}$$

By solving these equations, we obtain the Bloch modes and dispersion relation of the system. The dispersion relation is given by [13]

$$\frac{\Omega_0}{\pi} \cdot \frac{|r|^2}{2}[\cos(K_x\Lambda - 2\pi\phi_x) + \cos(K_y\Lambda - 2\pi\phi_y)] = -(\omega - \omega_0)[1 + O(|r|^2)], \tag{21}$$

where $\Omega_0 = 2\pi\frac{c}{S_c}$ is the free spectral range of the main cavity. Since the coupling is weak, $|r|^2 \ll 1$, we can drop the higher order correction term $O(|r|^2)$. Thus, from the dispersion relation in Eq. (21) and the tight-binding Hamiltonian in Eq. (13), we get

$$\kappa = \frac{\Omega_0}{\pi} \cdot \frac{|r|^2}{4}. \tag{22}$$

Gauge transformation

It is well known that a magnetic field can be described by different vector potentials which are related by a gauge transformation. This gauge transformation can be implemented and tested in our system. As depicted in Supplementary Figure 2 (a), we balance the lengths of the two optical paths in the auxiliary cavities that contain the SLMs, and insert a pair of beam rotators (BRs) with opposite rotation angles $\pm\vartheta = \pm 2\pi\phi_0$ in the two arms of the coupling cavities. The design of the BRs is shown in Supplementary Figure 3, where Dove prisms, that flip the transverse profile of any transmitted beam [14, 15], are used. By changing the azimuthal phase dependence of the l-th OAM mode from $e^{il\varphi}$ to $e^{il(\varphi \pm 2\pi\phi_0)}$, they cause a phase shift of $e^{\pm i2\pi l\phi_0}$ in the wave function when a photon tunnels between two adjacent cavities. The simulated Hamiltonian then becomes

$$\mathcal{H}_2 = -\kappa \sum_{j,l} \left(a^\dagger_{j,l+1} a_{j,l} + a^\dagger_{j,l} a_{j,l+1} + e^{-i2\pi l\phi_0} a^\dagger_{j+1,l} a_{j,l} + e^{i2\pi l\phi_0} a^\dagger_{j,l} a_{j+1,l} \right), \tag{23}$$

which is a *2d* system in a magnetic field with ϕ_0 quanta of flux per plaquette. $\mathcal{H}_2$ in Eq. (23) is related to $\mathcal{H}_1$ in Eq. (14) by a gauge transformation.

Though $\mathcal{H}_2$ and $\mathcal{H}_1$ describe the same physics since they are related by a gauge transformation, their implication for and requirement on the simulation system can be quite different. When we are interested in bulk properties (see Supplementary Note 6), a minimum number of unit cells in the simulated 2d system are needed. Interestingly, this places different requirements on the number of sites in both directions. It is because, for a rational magnetic flux $\phi_0 = p/q$ (p and q mutually prime integers), the size of the magnetic unit cell is $1 \times q$. Consequently, the system has a period of 1 in one direction and q in the other. Therefore, to simulate a system with $M \times M$ magnetic unit cells, the size of the simulated system should be $M \times qM$. Obviously, since the sizes in both directions are different, we should choose a gauge in which the larger dimension is represented with the degree of freedom that supports more sites. In our system, the number of OAM modes in a cavity is much larger than the number of cavities that can be coupled. This means that we should choose $\mathcal{H}_2$ to minimize the size of the simulator (see Supplementary Note 6). It requires M cavities for simulating a system containing $M \times M$ magnetic unit cells, whereas qM cavities would have been needed if $\mathcal{H}_1$ was chosen. As can be seen in this example, though $\mathcal{H}_2$ and $\mathcal{H}_1$ are related by a gauge transformation

and describe the same physics, there is a major difference from the simulation point of view.

Characteristics of the simulated system in the x and y direction

The characteristics of our simulated 2d systems are very different in the x and y direction because they are represented by completely different degrees of freedom. In the y direction, the sites of the lattice correspond to OAM modes in the same cavity. Theoretically, since there is no upper limit for the OAM of photons, the dimension in the y direction is infinite. In practice, properly designed degenerate cavities can accommodate many OAM modes, making the number of sites in the y direction very large. As can be seen from Supplementary Figure 2 (a), neighboring OAM states in the same cavity are coupled by the same set of BSs. Consequently, the coupling strengths between them are all equal in theory. This is a huge advantage, and much better uniformity along the y direction can be achieved than what is possible in a chain of coupled individual cavities whose sizes and separations will inevitably have errors.

In the x direction, multiple cavities need to be coupled in a chain. If conventional optical cavities of macroscopic sizes are used at visible and near-infrared wavelengths, the fluctuation in their lengths caused by thermal noise and other disturbances can be comparable to the wavelength and it is difficult to couple a large number of cavities. Nevertheless, because of the importance of laser phase and frequency stabilization in many contexts, there has been a long history of development of experimental techniques to deal with this problem [16]. By using advanced experimental techniques, it is now possible to lock multiple cavities and perform sophisticated experiments [17, 18]. As shown in the main text, to observe and study topological effects in our system, we only need a small 1d array with just a few cavities which is within the capability of current technologies. To increase the number of cavities that can be coupled, one can use technologies with more stable cavities, or work with photons with longer wavelengths such as microwave or maser photons [19, 20].

Another issue in the x direction is with the coupling strength between cavities. Since all OAM modes in the same cavity are eigen solutions of the same wave equation, once 1 OAM mode in a cavity is locked with the corresponding mode in the neighboring cavity, all other OAM modes are locked too. Therefore, locking cavities with multiple OAM modes is not more difficult than locking cavities with a single mode only. Still, coupling strengths between different cavities can fluctuate since they are realized with different optical elements. Such fluctuations in the coupling strength between cavities have an adverse impact on propagation of light through the body of the simulated lattice by in-band bulk states, but they obviously do not disturb the edge-state transport which is confined to the edge of the system. This is true as long as these fluctuations are much smaller than the band gap of the system and do not destroy its topology, a requirement not difficult to meet because of the availability of BSs with very accurate reflectivities. To see quantitatively how the simulation is affected by errors in the coupling strength, we plot the average OAM displacement (which is defined in equation (39) and shown to be determined by the Chern number of the system) for the photon transmission and its fluctuation caused by such errors in Supplementary Figure 4 (a). It can be seen that edge-state transport in the band gaps is hardly disturbed by small errors in the coupling strength between cavities.

OAM-Dependence of the tunneling coefficient and photon loss

As mentioned above, the couplings between different OAM states in the same cavity are realized with the same set of BSs and thus in principle they should all be equal. This argument is complicated by the practical consideration that, in reality, the SLMs have only limited resolution, and couplings between OAM modes can be dependent on the OAM number l because their spatial extends are different, especially for high OAM modes. This is only an issue when the photon loss is very low (otherwise very little light propagates to high OAM modes). It can be dealt with by using high-resolution SLMs for which such dependence is very weak. There are also experimental techniques to minimize and eliminate such dependence. For instance, it is experimentally demonstrated in [21] that the spatial extends of the OAM modes can be made the same on two SLMs in the optical path provided that appropriate optical design is used between them to place them in each other's near fields. Similar techniques can be used in our system to design the round-trip ray matrix such that the spatial extends of the OAM modes return to their original value when they come back to the SLM after a round-trip in the cavity following an increment/decrement in their OAM number by the SLM.

Nevertheless, considering the many inevitable and uncontrollable uncertainties in an actual experiment, the couplings between high OAM modes will likely have some, albeit weak dependence on the OAM number despite the precautions taken. The quality factors of the high OAM modes can depend on the mode number too, since modes with different spatial extends will have different leakage. Due to this OAM dependence, the characteristics of the component related uncertainties in our system are different than those in a *2d* cavity array where they are independent for each cavity. Assuming the same magnitude for the uncertainties in each case (though in reality the uncertainties

in a *2d* cavity array are likely much greater when the size of the array is large), this distinction in their characteristics should be insignificant, because topological protection ensures that edge-state transport is not disturbed by the uncertainties as long as they are much smaller than the band gap of the system and thus do not destroy its topology. Though the exact dependence on the OAM number is difficult to calculate, in a numeric simulation to check the robustness of the edge-state transport we can assume any dependence since topological protection is not sensitive to the exact form of the local noise. In Supplementary Figure 4 (b), we show the calculated average OAM displacement for an ideal system without uncertainties and its fluctuations caused by errors in the coupling strength and Q factors, assuming a particular dependence on the OAM number which results in larger errors for higher OAM modes. As we can see, within the band gaps where the transport is via edge states, the average OAM displacement is hardly disturbed by the OAM dependent errors. In contrast, the in-band bulk state transport is strongly affected. For comparison, we perform the same calculation for a *2d* cavity array and plot the results in Supplementary Figure 4 (c), by assuming the same magnitude of errors in the parameters though they are independent for each cavity. As far as edge-state transport is concerned, there is no appreciable difference between the two cases. Therefore, though in reality the component related uncertainties in a large 2d cavity array are likely to be much greater than in our system, under the assumption of similar magnitude for the uncertainties the behavior of edge-state transport is the same.

Supplementary Note 3: Simulation system for non-Abelian gauge fields

In order to simulate topological physics associated with non-Abelian gauge fields, we use polarized photons and represent the spin up and down states with the horizontal ($|\leftrightarrow\rangle$) and vertical ($|\updownarrow\rangle$) polarization. The photon modes are $\hat{\mathbf{a}}^{\dagger}_{j,l} = (\hat{a}^{\dagger}_{j,l,\leftrightarrow}, \hat{a}^{\dagger}_{j,l,\updownarrow})$, where $\hat{a}^{\dagger}_{j,l,\leftrightarrow}$ and $\hat{a}^{\dagger}_{j,l,\updownarrow}$ are the creation operators for horizontally and vertically polarized photons at site (j,l).

The design of the main cavities of the simulator does not require any modification. The auxiliary and coupling cavities, however, need to be augmented with polarization manipulating elements. Shown in Supplementary Figure 5 (a) and (b) are birefringent waveplates used in the auxiliary and coupling cavities. Such wave plates can alter the polarization state of the photons because polarization components along the fast and slow axis travel at different speeds [10]. In Supplementary Figure 5 (a), when the fast axis of the waveplate aligns with the vertical polarization of the incident photons, the two polarization states acquire different phases after the photons pass through the waveplate [10],

$$\begin{pmatrix} |\leftrightarrow\rangle \\ |\updownarrow\rangle \end{pmatrix} \Rightarrow e^{i2\pi\phi\sigma_z} \begin{pmatrix} |\leftrightarrow\rangle \\ |\updownarrow\rangle \end{pmatrix}, \tag{24}$$

where σ_z is the Pauli matrix, and $e^{i2\pi\phi\sigma_z}$ is the corresponding Jones matrix with the phase ϕ dependent on the thickness of the waveplate. If the fast axis is rotated by 45° with respect to the vertical polarization of the incident photons as in Supplementary Figure 5 (b), the corresponding Jones matrix becomes $e^{i2\pi\phi\sigma_x}$. Likewise, by taking advantage of the fact that left and right-handed circularly polarized light travels at different speed in optical media with circular birefringence, we can design a polarization rotator which has a Jones matrix $e^{i2\pi\phi\sigma_y}$ [10]. More generally, with a proper combination of waveplates and (or) rotators, we can realize any desired Jones matrix $e^{i2\pi\phi\sigma_{\mathbf{n}}}$ [10], where $\sigma_n = \vec{\sigma}\cdot\mathbf{n}$ and $\mathbf{n} = (n_x, n_y, n_z)$ is an arbitrary unit vector.

In Supplementary Figure 5 (c), we design the coupling cavities in the x direction such that the optical paths $\mathrm{BS}_2^j \to \mathrm{BS}_4^{j+1}$ and $\mathrm{BS}_4^{j+1} \to \mathrm{BS}_2^j$ contain phases $\frac{kS_{\mathrm{a}}}{2} \pm 2\pi\phi_x$ and Jones matrices $e^{\pm i2\pi\alpha\sigma_1}$, where $\sigma_1 = \vec{\sigma}\cdot\mathbf{n_1}$ with $\mathbf{n_1}$ an arbitrary unit vector. The Hamiltonian of the coupling term in the x direction then reads

$$-\kappa\sum_{j,l}\left(\hat{\mathbf{a}}^{\dagger}_{j+1,l} e^{i2\pi(\phi_x+\alpha\sigma_1)}\hat{\mathbf{a}}_{j,l} + h.c.\right). \tag{25}$$

The physical meaning of the phases is easier to understand if we switch to the eigen polarization states of σ_1, $|\leftrightarrow'\rangle$ and $|\updownarrow'\rangle$. In these bases, Eq. (25) is

$$-\kappa\sum_{j,l}\left(e^{i2\pi(\phi_x-\alpha)}\hat{a}^{\dagger}_{j+1,l,\leftrightarrow'}\hat{a}_{j,l,\leftrightarrow'} + e^{i2\pi(\phi_x+\alpha)}\hat{a}^{\dagger}_{j+1,l,\updownarrow'}\hat{a}_{j,l,\updownarrow'} + h.c.\right). \tag{26}$$

Obviously, $2\pi(\phi_x \pm \alpha)$ are the tunneling phases for photons in states $|\leftrightarrow'\rangle$ and $|\updownarrow'\rangle$ respectively.

The design of the polarization manipulating circuits for the auxiliary cavities is shown in Supplementary Figure 5

(d). The tight-binding Hamiltonian for the system is then

$$\begin{aligned}\mathcal{H} = & -\kappa \sum_{j,l} \left(\hat{\mathbf{a}}^{\dagger}_{j+1,l} e^{i2\pi\hat{\theta}_x} \hat{\mathbf{a}}_{j,l} + \hat{\mathbf{a}}^{\dagger}_{j,l+1} e^{i2\pi\hat{\theta}_y} \hat{\mathbf{a}}_{j,l} + h.c. \right) \\ & + \sum_{j,l} \lambda_j \hat{\mathbf{a}}^{\dagger}_{j,l} \hat{\mathbf{a}}_{j,l},\end{aligned} \tag{27}$$

where λ_j is the detuning of the j-th cavity, and the tunneling phases are

$$\hat{\theta}_x = \phi_x + \alpha\sigma_1, \hat{\theta}_y = \phi_y + \beta\sigma_2, \tag{28}$$

with $\sigma_2 = \vec{\sigma} \cdot \mathbf{n_2}$ and $\mathbf{n_2}$ a unit vector. $2\pi\phi_x$, $2\pi\phi_y$ are the spin-independent part of the gauge fields.

The spin-dependent $\hat{\theta}_x$ and $\hat{\theta}_y$ in Eq. (28) do not necessarily commute [22]. When $\hat{\theta}_x\hat{\theta}_y \neq \hat{\theta}_y\hat{\theta}_x$, they correspond to non-Abelian gauge potentials, and the Hamiltonian in Eq. (27) can be used to simulate the effects of non-Abelian gauge fields.

Notice that the horizontal and vertical polarizations of light used in our simulation system are both clockwise circulating cavity modes. By assuming that there is no coupling between the clockwise and counterclockwise cavity modes, and restricting ourselves to clockwise cavity modes only, we can describe the behavior of the horizontal and vertical polarizations with the non-Abelian Hamiltonian in equation (27). Since the Jones matrix description applies to polarizations of light traveling in one direction, and we make use of clockwise cavity modes only, we are not simulating the physical time-reversal symmetry directly. Nevertheless, due to the optical setup of the system, the phases acquired by and transitions between vertical and horizontal polarizations are the same with those of spin up and down in an electronic system described by the Hamiltonian in equation (27). Because of this, we can have polarized photon edge states in our system which are topologically protected by the symmetry in the optical design for the two polarizations though they are not physical time-reversal conjugates.

Supplementary Note 4: Input-output formalism for photon transmission measurement

As described in the main text of the paper, we probe our system by coupling a light beam with a definitive OAM number (and polarization in studies associated with non-Abelian gauge fields) to a cavity in the simulator array and measuring the photon transmission to other OAM modes (and polarizations when relevant) in different cavities. To study the characteristics of the measured quantity, we now consider the transmission coefficient taking into account the effect of photon loss. The photon loss can be understood in terms of the coupling of the cavity modes with the outside world due to a coupling term $\mathcal{H}_{\text{INT}}$ in the system's total Hamiltonian

$$\mathcal{H} = \mathcal{H}_{\text{SYS}} + \mathcal{H}_{\text{BATH}} + \mathcal{H}_{\text{INT}}, \tag{29}$$

where $\mathcal{H}_{\text{SYS}}$, $\mathcal{H}_{\text{BATH}}$ are the Hamiltonian for the cavity field and outside bath field. In the rotated frame with respect to the resonance frequency of the cavity, we have

$$\mathcal{H}_{\text{BATH}} = \sum_n \int_{-\infty}^{+\infty} d\omega [\omega d^{\dagger}_n(\omega) d_n(\omega)], \tag{30}$$

and

$$\mathcal{H}_{\text{INT}} = -i \sum_n \int_{-\infty}^{+\infty} d\omega \sqrt{\frac{\gamma_n}{2\pi}} [d_n(\omega) a^{\dagger}_n - a_n d^{\dagger}_n(\omega)]. \tag{31}$$

Here, $n = [j, l, s]$ is a collection of quantum numbers to specify a cavity photon mode. It includes the index of the cavity in the simulator array (j), the OAM number (l) of the photon, and its polarization state ($s = \leftrightarrow, \updownarrow$) when relevant. ω denotes the frequency detuning from the resonance frequency ω_0. $d_n(\omega)$ is the operator for the environment field coupled to the cavity photon mode labeled by n. $d_n(\omega)$ obeys the commutation relation

$$[d_n(\omega), d^{\dagger}_{n'}(\omega')] = \delta_{nn'}\delta(\omega - \omega').$$

The system Hamiltonian has a bilinear form

$$\mathcal{H}_{\rm SYS} \;=\; \sum_{n,n'} a_n^\dagger H_{nn'} a_{n'}, \tag{32}$$

where $H_{nn'}$ is the matrix element of the simulated Hamiltonian $\mathcal{H}_{\rm SYS}$.

Using the input-output theorem [23], we can write the Langevin equation of the system operators,

$$\begin{aligned}\frac{da_n(t)}{dt} &= -i[a_n, \mathcal{H}_{\rm SYS}] - \frac{\gamma_n}{2} a_n(t) - \sqrt{\gamma_n} d_{{\rm in},n}(t) \\ &= -i\sum_{n'} H_{nn'} a_{n'}(t) - \frac{\gamma_n}{2} a_n(t) - \sqrt{\gamma_n} d_{{\rm in},n}(t),\end{aligned} \tag{33}$$

where $d_{{\rm in},n}(t) = \frac{1}{\sqrt{2\pi}} \int_{-\infty}^{+\infty} d\omega e^{-i\omega t} d_{n,0}(\omega)$ is the input field operator, with $d_{n,0}(\omega)$ the value of $d_n(\omega)$ at $t = 0$. The output field is obtained from the input-output formalism

$$d_{{\rm out},n}(t) - d_{{\rm in},n}(t) = \sqrt{\gamma_n} a_n(t).$$

Making a Fourier transformation, we get

$$\begin{aligned}-i\omega a_n(\omega) &= -i\sum_{n'} H_{nn'} a_{n'}(\omega) - \frac{\gamma_n}{2} a_n(\omega) - \sqrt{\gamma_n} d_{{\rm in},n}(\omega), \\ \sqrt{\gamma_n} a_n(\omega) &= d_{{\rm out},n}(\omega) - d_{{\rm in},n}(\omega).\end{aligned} \tag{34}$$

The solution is

$$d_{{\rm out},n'}(\omega) = \sum_n \left\{ \delta_{n'n} - i \left[\sqrt{\Gamma} \frac{1}{\omega - \mathcal{H}_{\rm SYS} + i\Gamma/2} \sqrt{\Gamma} \right]_{n'n} \right\} d_{{\rm in},n}(\omega), \tag{35}$$

where $\Gamma = {\rm diag}\{\gamma_1, \gamma_2, \gamma_3, \ldots\}$ is the decay matrix. The first term on the right hand side, $d_{{\rm in},n'}(\omega)$, is the reflection. The rest describes field transmission. The transmission coefficient is

$$T_n^{n'} = -i \left[\sqrt{\Gamma} \frac{1}{\omega - \mathcal{H}_{\rm SYS} + i\Gamma/2} \sqrt{\Gamma} \right]_{n'n}. \tag{36}$$

For the simple case when all cavity modes decay with the same rate $\gamma_n = \gamma$ ($\forall n$), the transmission coefficient is

$$T_n^{n'} = -i\langle n'| \frac{\gamma}{\omega - \mathcal{H}_{\rm SYS} + i\gamma/2} |n\rangle, \tag{37}$$

where $|n\rangle = \hat{a}_n^\dagger |0\rangle$ is a single photon state.

Supplementary Note 5: OAM displacement in edge-state transport

It is demonstrated in the main text that, when the frequency of a probing light falls in a gap in the spectrum of a finite *2d* lattice in magnetic field with the Hamiltonian

$$\mathcal{H} = -\kappa \sum_{j,l} \left(e^{i2\pi j\phi_0} \hat{a}_{j,l+1}^\dagger \hat{a}_{j,l} + \hat{a}_{j+1,l}^\dagger \hat{a}_{j,l}, +h.c. \right), \tag{38}$$

it can only propagate along the edge of the lattice because of edge-state excitation. We discovered a quantity that is very useful for the study of edge-state transport. It is the average OAM displacement defined as

$$\bar{l}_{\rm e} = \sum_{j\in {\rm edge}} \sum_{j_{\rm o}, l_{\rm o}} |T_{j,0}^{j_{\rm o}, l_{\rm o}}|^2 \cdot l_{\rm o}, \tag{39}$$

where $T_{j,0}^{j_{\rm o}, l_{\rm o}}$ is the photon transmission coefficient defined in Eq. (37) and $\sum_{j\in {\rm edge}}$ refers to summation over the region close to one edge (left or right) of the lattice where the amplitude of the corresponding edge states is appreciable.

It can be shown that $\bar{l}_e$ defined in Eq. (39) is related to the Chern number of the system. To prove this, we consider a system in the Laughlin-Halperin geometry which has open and periodic boundary condition in the x and y direction. In such a system, there are two sets of chiral edge states, one per boundary, that propagate in opposite directions [24, 25]. Consequently, the displacement $\bar{l}_e$ due to transport by edge states on the left and right edges are equal in magnitude but opposite in sign. Without loss of generality, we will focus on the left edge, and restrict the summation of j to the region near the left edge of the lattice. Because of the periodic boundary condition in the y direction, the Bloch momentum $k_y = 2\pi\frac{n_y}{N_y}$ is a good quantum number of the system, where $n_y = 0, 1, \ldots, N_y - 1$ and N_y is the number of sites in the y direction. We can use the momentum representation in the y direction, $\hat{a}^\dagger_{j,k_y} = \frac{1}{\sqrt{N_y}}\sum_l e^{ik_y l}\hat{a}^\dagger_{j,l}$, and introduce the single-particle eigenfunction

$$|\Psi_{k_y}\rangle = \sum_j \Psi_{j,k_y}\hat{a}^\dagger_{j,k_y}|0\rangle, \tag{40}$$

where Ψ_{j,k_y} satisfies [24]

$$-\kappa\left(\Psi_{j+1,k_y} + \Psi_{j-1,k_y}\right) - 2\kappa\cos(k_y - 2\pi j\phi_0)\Psi_{j,k_y} = E_{k_y}\Psi_{j,k_y} \tag{41}$$

with E_{k_y} the eigenenergy.

We can now express the photon transmission coefficient in terms of $|\Psi_{k_y}\rangle$,

$$T^{j_o,l_o}_{j,0} = -i\langle j_o, l_o|\sum_{\{|\Psi_{k_y}\rangle\}}\left(|\Psi_{k_y}\rangle\frac{\gamma}{\omega - E_{k_y} + i\gamma/2}\langle\Psi_{k_y}|\right)|j,0\rangle. \tag{42}$$

Clearly, only states with energies close to the probing light frequency ω have significant contribution to $T^{j_o,l_o}_{j,0}$. Because of this, when ω falls in the mid of a gap in the system spectrum and γ is much smaller than the corresponding band gap, we can include only the edge states in calculating $T^{j_o,l_o}_{j,0}$ in Eq. (42) since in-band states are far off resonance. Deep in the band gap, the dispersion relation of the edge states is linear in k_y [24]. Taking into account the possibility of multiple edge modes in the vicinity of ω, we have $E^m_{k_y} = \omega + v_m(k^m_y - \Bbbk^m_y)$, where v_m is the group velocity of the m-th edge mode and $\Bbbk^m_y$ is the Bloch momentum of the state in resonance with the probing light ($E^m_{\Bbbk^m_y} = \omega$). Making use of the dispersion relation, we obtain in the continuum limit $N_y \to \infty$

$$T^{j_o,l_o}_{j,0} \simeq \frac{1}{2\pi}\sum_m\int dk^m_y\Psi^m_{j_o,k_y}\frac{i\gamma}{(k^m_y - \Bbbk^m_y)v_m - i\gamma/2}\Psi^{m*}_{j,k_y}e^{ik^m_y l_o}, \tag{43}$$

where Ψ^m is the m-th edge mode. Since only states close to $\Bbbk^m_y$ contribute to the integration in Eq. (43), we can evaluate it by approximating $\Psi^m_{j_o,k_y}$ with $\Psi^m_{j_o,\Bbbk^m_y}$ and extending the limit of the integration to $(-\infty,\infty)$. The result is

$$T^{j_o,l_o}_{j,0} \simeq \frac{1}{2\pi}\sum_m\Psi^m_{j_o,\Bbbk^m_y}\Psi^{m*}_{j,\Bbbk^m_y}\int_{-\infty}^{\infty}dk^m_y\frac{i\gamma}{(k_y - \Bbbk^m_y)v_m - i\gamma/2}e^{ik^m_y l_o} = -\sum_m\Psi^m_{j_o,\Bbbk^m_y}\Psi^{m*}_{j,\Bbbk^m_y}\frac{\gamma}{v_m}\Theta\left(\frac{l_o}{v_m}\right)e^{-\frac{\gamma}{2}\frac{l_o}{v_m}}e^{i\Bbbk^m_y l_o} \tag{44}$$

with the step function

$$\Theta(x) = \begin{cases} 0 & x < 0 \\ \frac{1}{2} & x = 0 \\ 1 & x > 0. \end{cases}$$

By using Eq. (44), it is straightforward to calculate the average OAM number displacement. We obtain

$$\bar{l}_e = \sum_{j\in\text{edge}}\sum_{j_o,l_o}|T^{j_o,l_o}_{j,0}|^2\cdot l_o \simeq \sum_{m\in\text{left}}\text{sgn}(v_m), \tag{45}$$

where the summation over m includes only the corresponding edge states on the left edge of the lattice. We have used $\sum_{j\in\text{edge}}|\Psi^m_{j,\Bbbk^m_y}|^2 \simeq 1$ when the m-th edge mode is on the left edge and $\sum_{j\in\text{edge}}|\Psi^m_{j,\Bbbk^m_y}|^2 \simeq 0$ when it is on the right edge, which follows from the fact that the distribution of the edge states is limited to the edge of the lattice. This result indicates that $\bar{l}_e$ is approximately equal to the difference between the number of up and down moving

edge states, which in turn is equal to the total Chern number (up to a sign depending on edge transport of the left or right edge) for the bands below the gap due to the bulk-boundary correspondence [26].

Supplementary Note 6: Measurement of the Chern number

As shown in the main text, the Chern number of a finite lattice can be measured via the average OAM number displacement ($\bar{l}_{\rm e}$ in Eq. (39)) in edge-state transport. For an infinite system, the Chern number is equal to the TKNN index [25, 27, 28]. We demonstrate in this section that it can be calculated from experimentally measured photon transmission coefficients.

The TKNN index in an infinite system is determined by the bulk wave function. As discussed in Supplementary Note 2, in order to keep the size of the simulator array small, we should choose a gauge that leads to the Hamiltonian

$$\mathcal{H}_2 = -\kappa \sum_{j,l} \left(a^\dagger_{j,l+1} a_{j,l} + a^\dagger_{j,l} a_{j,l+1} + e^{-i2\pi l \phi_0} a^\dagger_{j+1,l} a_{j,l} + e^{i2\pi l\phi_0} a^\dagger_{j,l} a_{j+1,l} \right), \tag{46}$$

where $\phi_0 = p/q$ (p and q mutually prime integers) is the flux quanta per plaquette. The configuration of the simulation system has been described in Supplementary Note 2.

We use periodic boundary condition in both the x and y directions to simulate an infinite system. According to the Bloch theorem, the eigenstates of $\mathcal{H}_2$ can be written in the form

$$\Psi_{j,l}(k_x, k_y) = e^{ik_y l} e^{ik_x j} u_{l_q}(k_x, k_y), \tag{47}$$

where $k_x \in [-\pi, \pi]$, $k_y \in [0, 2\pi/q]$ are the Bloch vectors, $l_q = \mathrm{mod}(l, q) \in [0, q-1]$ is the OAM index within a magnetic unit cell, and $u_{l_q}(k_x, k_y) = u_{l_q+q}(k_x, k_y)$ is a periodic function.

The spectrum of the system consists of q energy bands [29]. The Chern number (or equivalently the TKNN index) of the m-th ($m \in [1, q]$) band can be expressed as [25, 27, 28]

$$C = \frac{1}{2\pi i} \int\int dk_x dk_y (\langle \frac{\partial u^m}{\partial k_x} | \frac{\partial u^m}{\partial k_y} \rangle - \langle \frac{\partial u^m}{\partial k_y} | \frac{\partial u^m}{\partial k_x} \rangle) = \frac{1}{2\pi i} \int\int dk_x dk_y [\nabla_k \times \mathbf{A}^m(k_x, k_y)]_z, \tag{48}$$

where $\mathbf{A}^m = \langle u^m | \nabla_k | u^m \rangle$ and

$$|u^m(k_x, k_y)\rangle = [u_0^m(k_x, k_y), \ldots, u_{q-1}^m(k_x, k_y)]^{\rm T} \tag{49}$$

is the eigenstate vector of the m-th band. There is a gauge freedom which comes from the phase ambiguity of $|u^m(k_x, k_y)\rangle$, since

$$e^{if(k_x,k_y)} |u^m(k_x, k_y)\rangle \tag{50}$$

is also a solution as long as $f(k_x, k_y)$ is a smooth function of (k_x, k_y) and it is independent of (x, y). The Chern number is invariant under this gauge transformation.

A non-trivial topology arises when the phase of the wave function cannot be determined uniquely and smoothly in the entire magnetic Brillouin zone. In this case, one cannot apply the Stokes theorem globally to evaluate Eq. (48) [28]. Following Refs. [25, 28], we divide the Brillouin zone into two regions B1 and B2 [see Supplementary Figure 6 (b)], where B2 is chosen such that it contains all zero points of $u_0^m(k_x, k_y)$ and at least one $u_{l_q}^m(k_x, k_y)$ with $l_q \neq 0$ does not vanish in it. By taking advantage of the gauge transformation in Eq. (50) with an appropriate $f(k_x, k_y)$, we can choose a phase convention in B1 such that $u_0^m(k_x, k_y)$ is real, and another phase convention in B2 such that $u_{l_q}^m(k_x, k_y)$ is real. The chosen phase conventions lead to smooth vector fields $\mathbf{A}^m_{\rm B1}$ and $\mathbf{A}^m_{\rm B2}$ on B1 and B2 respectively, and result in a phase mismatch $\chi(k_x, k_y)$ on the boundary of B1 and B2 [28],

$$|u^m\rangle_{\rm B1} = e^{i\chi(k_x,k_y)} |u^m\rangle_{\rm B2}. \tag{51}$$

We can then apply Stokes' theorem on B1 and B2 separately to derive

$$C = \frac{1}{2\pi i} \int_{\partial \rm B1} d\mathbf{k} \cdot [\mathbf{A}^m_{\rm B1}(k_x, k_y) - \mathbf{A}^m_{\rm B2}(k_x, k_y)] = \frac{1}{2\pi} \int_{\partial \rm B1} d\mathbf{k} \cdot \nabla_k \chi(k_x, k_y), \tag{52}$$

where ∂B1 is the boundary of B1.

We can obtain $|u^m\rangle$ and determine $\chi(k_x, k_y)$ from photon transmission measurement and then use Eq. (52) to calculate the Chern number. Suppose we couple a $l = 0$ OAM beam to the first cavity in the simulator array, which is equivalent to driving the simulated lattice system at site $(0, 0)$, and measure the transmission coefficient to site (j, l), $T_{0,0}^{j,l}$. The Fourier transformation of $T_{0,0}^{j,l}$ to the momentum space (k_x, k_y), $T(k_x, k_y, l_q) \propto \sum_{j,l} T_{0,0}^{(j,ql+l_q)} e^{-ik_x j} e^{-ik_y(ql+l_q)}$, is given by

$$T(k_x, k_y, l_q) \propto \langle k_x, k_y, l_q| \frac{i\gamma}{\omega - \mathcal{H} + i\gamma} |j = 0, l = 0\rangle \quad (53)$$

where $|k_x, k_y, l_q\rangle \propto \sum_{j,l} e^{ik_x j} e^{ik_y(ql+l_q)} |j, ql + l_q\rangle$. If the photon loss rate γ is much smaller than the band gaps, and the driving frequency is close to the m-th band, only states in the m-th band are excited and contribute to the transmission. Consequently,

$$T(k_x, k_y, l_q) \propto u_{l_q}^m(k_x, k_y) \frac{i\gamma}{\omega - E_m(k_x, k_y) + i\gamma} u_0^m(k_x, k_y)^*, \quad (54)$$

where $E_m(k_x, k_y)$ is the energy of the m-th band at (k_x, k_y). By using a similar idea in [30], for each (k_x, k_y) we can fine tune the driving frequency such that it is in resonance with $E_m(k_x, k_y)$, i.e. $\omega - E_m(k_x, k_y) \ll \gamma$. This then allows us to relate the photon transmission coefficient to the wave function in the m-th band via

$$T(k_x, k_y, l_q) \propto u_{l_q}^m(k_x, k_y) u_0^m(k_x, k_y)^*. \quad (55)$$

By using Eq. (55) and renormalizing the measured $T(k_x, k_y, l_q)$, we can determine the eigenstate $|u^m(k_x, k_y)\rangle = [u_0^m(k_x, k_y), u_1^m(k_x, k_y), \ldots, u_{q-1}^m(k_x, k_y)]^{\mathrm{T}}$ of the m-th band. With the help of the gauge transformation in Eq. (50), we can further choose the phase of the eigenstate $|u^m\rangle$ in the magnetic Brillouin zone using the technique discussed earlier. This then allows us to determine $\chi(k_x, k_y)$ in Eq. (51) and calculate the Chern number according to Eq. (52).

As an example, we consider the flux $p/q = 1/6$, and show how to measure the Chern number of the first band ($m = 1$). From the band structure in Supplementary Figure 6 (a), we see that this band is located near $\omega = -3.09\kappa$ and it is very narrow. With a photon loss rate of $\gamma = 0.1\kappa$, which is much larger than the width of this band and much smaller than the band gaps surrounding it, we can achieve resonance with all states in it and avoid exciting states in other bands by fixing the frequency of the probing light at $\omega = -3.09\kappa$.

We then divide the magnetic Brillouin zone into two areas as prescribed earlier. Specifically, we define $B1 = \{k_x \in [-0.4\pi, 0.4\pi], ky \in [0, 2\pi/q]\}$, and the rest B2, as depicted in Supplementary Figure 6 (b). In B1, $u_0^1(k_x, k_y)$ is always nonzero. B2 contains all the zero points of $u_0^1(k_x, k_y)$. Also, u_3^1 does not vanish in B2. As discussed earlier, with this division we can define two different phase conventions for the eigenstates in B1 and B2 [25, 28]. In one convention, $u_0^1(k_x, k_y)$ is real in B1. In the other convention, $u_3^1(k_x, k_y)$ is real in B2. From Eq. (51), we see that the phase mismatch $\chi(k_x, k_y)$ on the boundary ∂B1 is given by the phase of $u_3^1(k_x, k_y)$ on ∂B1. According to Eq. (55), if we drive the simulated system at site $(0, 0)$, we have $T(k_x, k_y, l_q) \propto u_{l_q}^1(k_x, k_y) u_0^1(k_x, k_y)^*$, from which we can obtain $|u^1\rangle \propto [T(k_x, k_y, 0), T(k_x, k_y, 1), \ldots, T(k_x, k_y, 5)]^{\mathrm{T}}$. Therefore, $\chi(k_x, k_y)$ is given by the phase of $T(k_x, k_y, 3)$ relative to that of $T(k_x, k_y, 0)$ on ∂B1, boundary of B1, and the Chern number can be calculated using Eq. (52).

Supplementary References

[1] Norman Hodgson and Horst Weber. *Laser resonators and beam propagation.* Springer New York, 2005.

[2] Jr Collins, A Stuart, et al. Lens-system diffraction integral written in terms of matrix optics. *JOSA*, 60(9):1168–1177, 1970.

[3] JA Arnaud. Degenerate optical cavities. *Applied optics*, 8(1):189–195, 1969.

[4] Sylvain Gigan, Laurent Lopez, Nicolas Treps, Agnès Maître, and Claude Fabre. Image transmission through a stable paraxial cavity. *Physical Review A*, 72(2):023804, 2005.

[5] Benoît Chalopin, Antonino Chiummo, Claude Fabre, Agnès Maître, and Nicolas Treps. Frequency doubling of low power images using a self-imaging cavity. *Optics express*, 18(8):8033–8042, 2010.

[6] L Allen, MW Beijersbergen, RJC Spreeuw, and JP Woerdman. Orbital angular momentum of light and the transformation of laguerre-gaussian laser modes. *Physical Review A*, 45(11):8185, 1992.

[7] Joyce KS Poon, Jacob Scheuer, Yong Xu, and Amnon Yariv. Designing coupled-resonator optical waveguide delay lines. *JOSA B*, 21(9):1665–1673, 2004.

[8] Mehmet Bayindir, B Temelkuran, and E Ozbay. Tight-binding description of the coupled defect modes in three-dimensional photonic crystals. *Physical Review Letters*, 84(10):2140, 2000.

[9] Michael J Hartmann, Fernando GSL Brandao, and Martin B Plenio. Strongly interacting polaritons in coupled arrays of cavities. *Nature Physics*, 2(12):849–855, 2006.

[10] Amnon Yariv and Pochi Yeh. *Photonics: Optical Electronics in Modern Communications.* Oxford University Press, Oxford, 2007.

[11] Ioannis Chremmos and Nikolaos Uzunoglu. Propagation in a directional coupler of parallel microring coupled-resonator optical waveguides. *Optics Communications*, 281(12):3381–3389, 2008.

[12] Ioannis Chremmos and Nikolaos Uzunoglu. Modes of the infinite square lattice of coupled microring resonators. *JOSA A*, 25(12):3043–3050, 2008.

[13] Mohammad Hafezi, Eugene A Demler, Mikhail D Lukin, and Jacob M Taylor. Robust optical delay lines with topological protection. *Nature Physics*, 7(11):907–912, 2011.

[14] Jonathan Leach, Miles J Padgett, Stephen M Barnett, Sonja Franke-Arnold, and Johannes Courtial. Measuring the orbital angular momentum of a single photon. *Physical review letters*, 88(25; PART 1):257901–257901, 2002.

[15] M BORN and E WOLF. *Principles of optics.* Cambridge University Press, Cambridge 1980.

[16] R. W. P. Drever, J. L. Hall, F. V. Kowalski J. Hough, G. M. Ford, A. J. Munley, and H. Ward, Laser phase and frequency stabilization using an optical resonator. *Appl. Phys. B: Photophys. Laser Chem.* 31:97–105, 1983.

[17] Xiaolong Su, Yaping Zhao, Shuhong Hao, Xiaojun Jia, Changda Xie, and Kunchi Peng. Experimental preparation of eight-partite cluster state for photonic qumodes. *Optics Letters*, vol 37, no 24, 5178, 2012.

[18] Shota Yokoyama et al. Ultra-large-scale continuous-variable cluster states multiplexed in the time domain. *Nature Photonics*, 7(5):982–986, 2013.

[19] F. Tamburini, E. Mari, B. Thidé, C. Barbieri, and F. Romanato, Experimental verification of photon angular momentum and vorticity with radio techniques, *Appl. Phys. Lett.* **99**, 204102 (2011).

[20] F. Tamburini, E. Mari, A. Sponselli, B. Thidé, A. Bianchini, and F. Romanato, Encoding many channels on the same frequency yhtough radio vorticity: first experimental test, *New J. Phys.* **12**, 033001 (2012).

[21] Oemrawsingh, S. S. R. *et al.* Production and characterization of spiral phase plates for optical wavelengths. *Appl. Opt.* **43,** 688–694 (2004).

[22] Jean Dalibard, Fabrice Gerbier, Gediminas Juzeliūnas, and Patrik Öhberg. Colloquium: Artificial gauge potentials for neutral atoms. *Reviews of Modern Physics*, 83(4):1523, 2011.

[23] DF Walls and Gerard J Milburn. *Quantum optics.* Springer-Verlag, Berlin, 2008.

[24] Yasuhiro Hatsugai. Edge states in the integer quantum hall effect and the riemann surface of the bloch function. *Physical Review B*, 48(16):11851, 1993.

[25] Yasuhiro Hatsugai. Chern number and edge states in the integer quantum hall effect. *Physical review letters*, 71(22):3697, 1993.

[26] M Zahid Hasan and Charles L Kane. Colloquium: topological insulators. *Reviews of Modern Physics*, 82(4):3045, 2010.

[27] DJ Thouless, M Kohmoto, MP Nightingale, and M Den Nijs. Quantized hall conductance in a two-dimensional periodic potential. *Physical Review Letters*, 49:405–408, 1982.

[28] Mahito Kohmoto. Topological invariant and the quantization of the hall conductance. *Annals of Physics*, 160(2):343–354, 1985.

[29] Douglas R Hofstadter. Energy levels and wave functions of bloch electrons in rational and irrational magnetic fields. *Physical review B*, 14(6):2239, 1976.

[30] Ozawa, T. and Carusotto, I. Anomalous and Quantum Hall Effects in Lossy Photonic Lattices. *Phys. Rev. Lett.* **112,** 133902 (2014).
